# Probabilistic Approach to Predicting Substrate Specificity of Methyltransferases

**DOI:** 10.1371/journal.pcbi.1003514

**Published:** 2014-03-20

**Authors:** Teresa Szczepińska, Jan Kutner, Michał Kopczyński, Krzysztof Pawłowski, Andrzej Dziembowski, Andrzej Kudlicki, Krzysztof Ginalski, Maga Rowicka

**Affiliations:** 1 Nencki Institute of Experimental Biology, Polish Academy of Sciences, Warsaw, Poland; 2 Department of Biochemistry and Molecular Biology, University of Texas Medical Branch, Galveston, Texas, United States of America; 3 Institute for Translational Sciences, University of Texas Medical Branch, Galveston, Texas, United States of America; 4 Institute of Biochemistry and Biophysics, Polish Academy of Sciences, Warsaw, Poland; 5 Institute of Genetics and Biotechnology, Faculty of Biology, University of Warsaw, Warsaw, Poland; 6 Laboratory of Bioinformatics and Systems Biology, Centre of New Technologies, University of Warsaw, Warsaw, Poland; 7 Warsaw University of Life Sciences, Warsaw, Poland; 8 Sealy Center for Molecular Medicine, University of Texas Medical Branch, Galveston, Texas, United States of America; Wellcome Trust Sanger Institute, United Kingdom

## Abstract

We present a general probabilistic framework for predicting the substrate specificity of enzymes. We designed this approach to be easily applicable to different organisms and enzymes. Therefore, our predictive models do not rely on species-specific properties and use mostly sequence-derived data. Maximum Likelihood optimization is used to fine-tune model parameters and the Akaike Information Criterion is employed to overcome the issue of correlated variables. As a proof-of-principle, we apply our approach to predicting general substrate specificity of yeast methyltransferases (MTases). As input, we use several physico-chemical and biological properties of MTases: structural fold, isoelectric point, expression pattern and cellular localization. Our method accurately predicts whether a yeast MTase methylates a protein, RNA or another molecule. Among our experimentally tested predictions, 89% were confirmed, including the surprising prediction that YOR021C is the first known MTase with a SPOUT fold that methylates a substrate other than RNA (protein). Our approach not only allows for highly accurate prediction of functional specificity of MTases, but also provides insight into general rules governing MTase substrate specificity.

## Author Summary

Our approach is easily applicable to different organisms, because it does not rely on species-specific properties and uses mostly sequence-derived and other readily available data (e.g. isoelectric point or predicted structural fold). Tests on yeast MTases indicate that the accuracy of our predictions is ∼90%. We show that knowledge of substrate binding sites or corresponding motifs is not crucial for highly accurate general substrate specificity predictions of enzymes, and provide new insights into how such specificities are achieved at the molecular level. We predict substrate specificities not yet observed for a given class of enzymes, and experimentally verify our predictions.

This is a *PLOS Computational Biology* Methods article.

## Introduction

Prediction of protein function from its sequence is an important goal of bioinformatics [1], [2], since the function of many proteins remains unknown, including more than 50% of human proteins. Because of its importance, a large-scale community-based Critical Assessment of Protein Function Annotation (CAFA) experiment is held biannually [3], to objectively evaluate and compare different methods and stimulate research in this area. One of the most difficult cases of protein function prediction is that of enzyme substrate specificity, which is essential for understanding its role in cellular processes. Even if the exact 3D structure of an enzyme is known, its substrate specificity is often not clear, as it depends on both local (e.g. active site) and global (e.g. protein structure) properties [2], [4], [5].

Many approaches have been proposed to predict enzyme substrate specificity. One example, applied to type II restriction endonucleases (REases), relied on the observation that connectivity of the secondary structures in the αβα structural core correlates with the angles between the secondary structure elements and the cleavage patterns of the REases [6]. Prediction of optimal substrate peptides (encompassing the phosphorylation site) for protein kinases was done taking only the amino acid sequence of a kinase as input [7]. Analysis of available crystal structures, molecular modeling, and sequence analyses of kinases and substrates led to extraction of a set of rules governing the substrate specificity of protein serine/threonine kinases. The method was used to analyze yeast cell cycle control and DNA damage checkpoint pathways. Combined genomic and functional context was recently used in Zhang *et al.* [8] to assign function of homologous proteins from the carbohydrate FGGY kinase family. However, homology alone is not sufficient to successfully predict protein substrate specificity [4], [9].

Several bioinformatics approaches have been applied to predict substrate specificity of yeast MTases. An attempt to infer the substrate of methylation from a hidden Markov model profile clustering analysis, applied to *Saccharomyces cerevisiae* Rossmann-like fold methyltransferases, revealed some grouping of MTases that correlated with their substrate specificity [10]. However, this method is limited and not capable of predicting substrate specificity for all studied proteins. In Wlodarski *et al.* [11] we proposed that fold, pI, temporal expression pattern and protein localization contribute to determining MTase substrate specificity.

The prediction methods discussed above typically rely on complex heuristics and in some cases require a detailed 3D structure of the protein, or are applicable only to some of the studied enzymes. Here, we propose a very general framework based on fundamental laws of probability that is applicable to all considered proteins (even in cases of missing data) and does not require any specific data type (e.g. known 3D structure, conserved sequence motifs). Moreover, our method is capable of correctly predicting substrate specificity from a combination of properties not yet observed among known enzymes. Our method has a much higher percentage of successful predictions (84–89%) than previous approaches and is not limited to a certain group of MTases [10], [11]. Since our approach is general and relies on features that are sequence-derived and not organism-specific, it should be easily applicable to other organisms and enzyme classes.

As proof-of-principle, our approach is employed to predict general substrate specificity of yeast MTases. MTases are present in all living organisms and involved in many important cellular processes such as signal transduction, transcriptional control, biosynthesis and metabolism [12]. MTases comprise a large and highly diverse group of enzymes that transfer a methyl group from a donor (typically S-Adenosyl-L-Methionine, SAM) to an acceptor (MTase substrate) [13]. In *S. cerevisiae*, there are 86 MTases and their substrates are either proteins, RNAs or other molecules, (DNA is not enzymatically methylated [14]) [11]. As a training set, we used 61 *S. cerevisiae* MTases with experimentally confirmed substrate specificity (known MTases) (Table S2) and predicted substrate specificities for 25 putative *S. cerevisiae* MTases with unknown substrate specificity (putative MTases). After our predictions were made, the substrate specificities of 9 MTases were confirmed experimentally, with results consistent with predictions in 89% (8 out of 9) of the cases.

## Results and Discussion

We propose a mathematical framework for inferring substrate specificities from the physico-chemical and biological properties of MTases. The advantage of our method is that it yields very accurate substrate specificity predictions and explicitly provides the probabilities that a given MTase methylates a substrate from each class (RNA, protein or other molecule). The method consists of three stages. First, we estimate conditional probability for each substrate specificity based on a single property. Second, the final probability is computed based on several selected properties. The single-property probabilities are combined as described in Materials And Methods. The high number of available enzyme properties leads to a very large combinatorial space of probabilistic models for predicting the substrate specificity. To limit the search for the best model, we selected the 22 most informative properties as defined by the likelihood of the respective single property models on the training set (Table S1). For numerical variables, we chose either continuous or binned representation, as well as optimal number of bins.

The final model is selected based on optimization of up to 14 parameters (Table S2) and evaluation of 86,000 models. Since the number of properties and range of parameters considered did not allow for an exhaustive search in the model space, we optimized continuous properties using the Powell method (Text S1. Supplementary text) [15]. Because we were comparing models with different numbers of parameters, the likelihood criterion would not be appropriate. Likelihood, which describes the goodness of fit, is always increased if more variables are added to the best performing model with a given number of parameters. Therefore, to compare models with differing numbers of parameters, we instead used the Akaike Information Criterion (AIC) [16], which balances the goodness of the fit (likelihood) with informativity of the parameters. The AIC naturally selects models using the most informative sets of parameters and rejects those with highly correlated parameters. This is important in our case, as we prefer to use parameters with clear biological or physico-chemical interpretation, which in general are not mutually independent.

### Probabilities of substrate specificities conditional on a single property

The probability of substrate specificity for an MTase with a certain property is given by the Bayes Theorem:
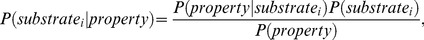
(1)where *P*(substrate*_i_*) is the probability of an MTase to methylate substrate type *i* (i.e. protein, RNA or other molecule) and *P*(property) is its probability to have a certain property (e.g. structural fold, isoelectric point (pI), expression pattern and cellular localization). *P*(substrate*_i_*|property) is the probability that an MTase will methylate substrate type *i* if this MTase has the given property. *P*(property|substrate*_i_*) is the probability of an MTase having a certain property if it methylates substrate type *i* and *P*(substrate*_i_*) is the a priori probability of substrate type *i*. A property can be either categorical (e.g. fold) or numerical (e.g. pI, expression onset), and in either case, a range of different predictive models can be constructed. To select the best single-property model, we apply the Maximum Likelihood (ML) method to optimize *P*(substrate*_i_*|property) on the training set (i.e. substrate specificities of the known MTases). The training set consists of 61 *S. cerevisiae* MTases with experimentally confirmed substrate specificity (known MTases) (Table S2). Among them 26 methylate RNAs, 24 methylate proteins and 11 methylate other molecules.

### Properties used as predictors in the model

**Figure 1:**
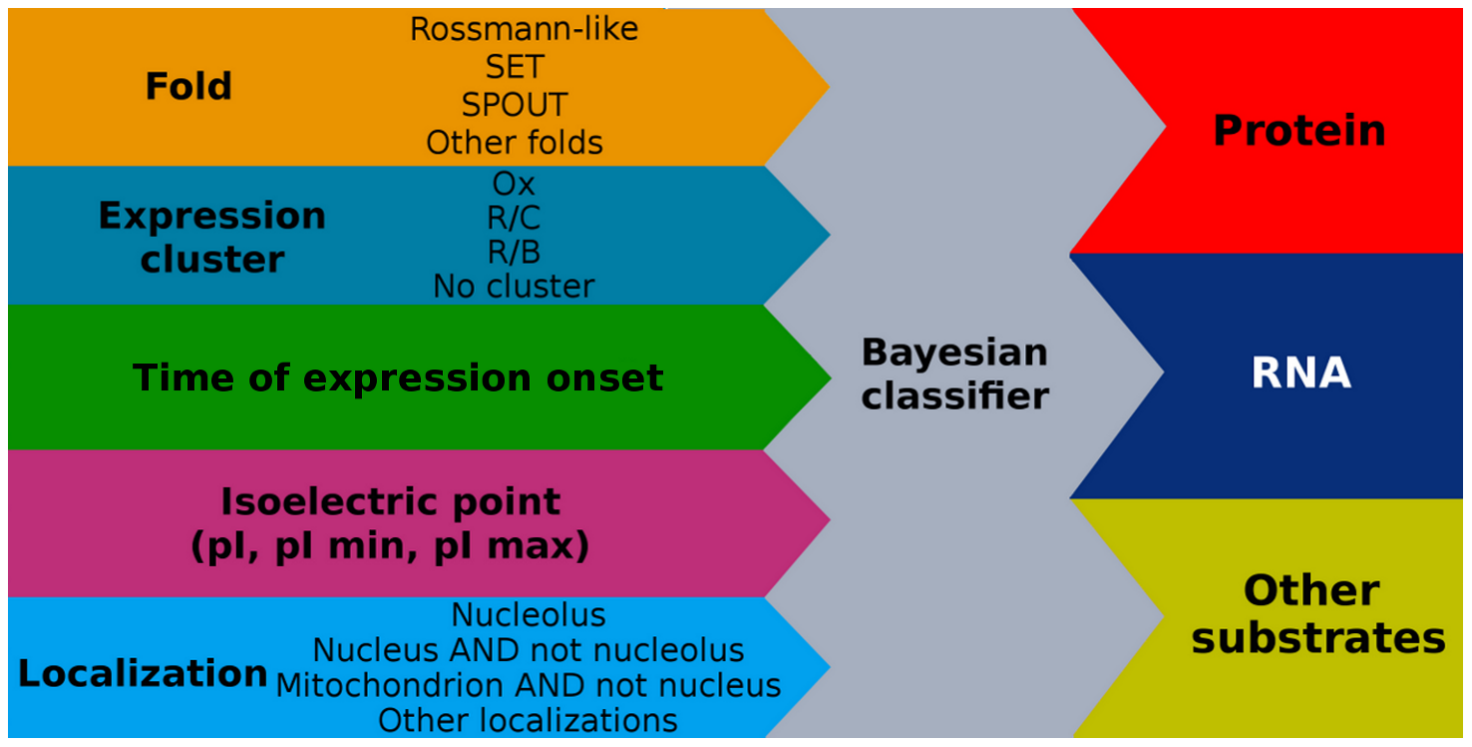
Workflow of the prediction model.

**Figure 2:**
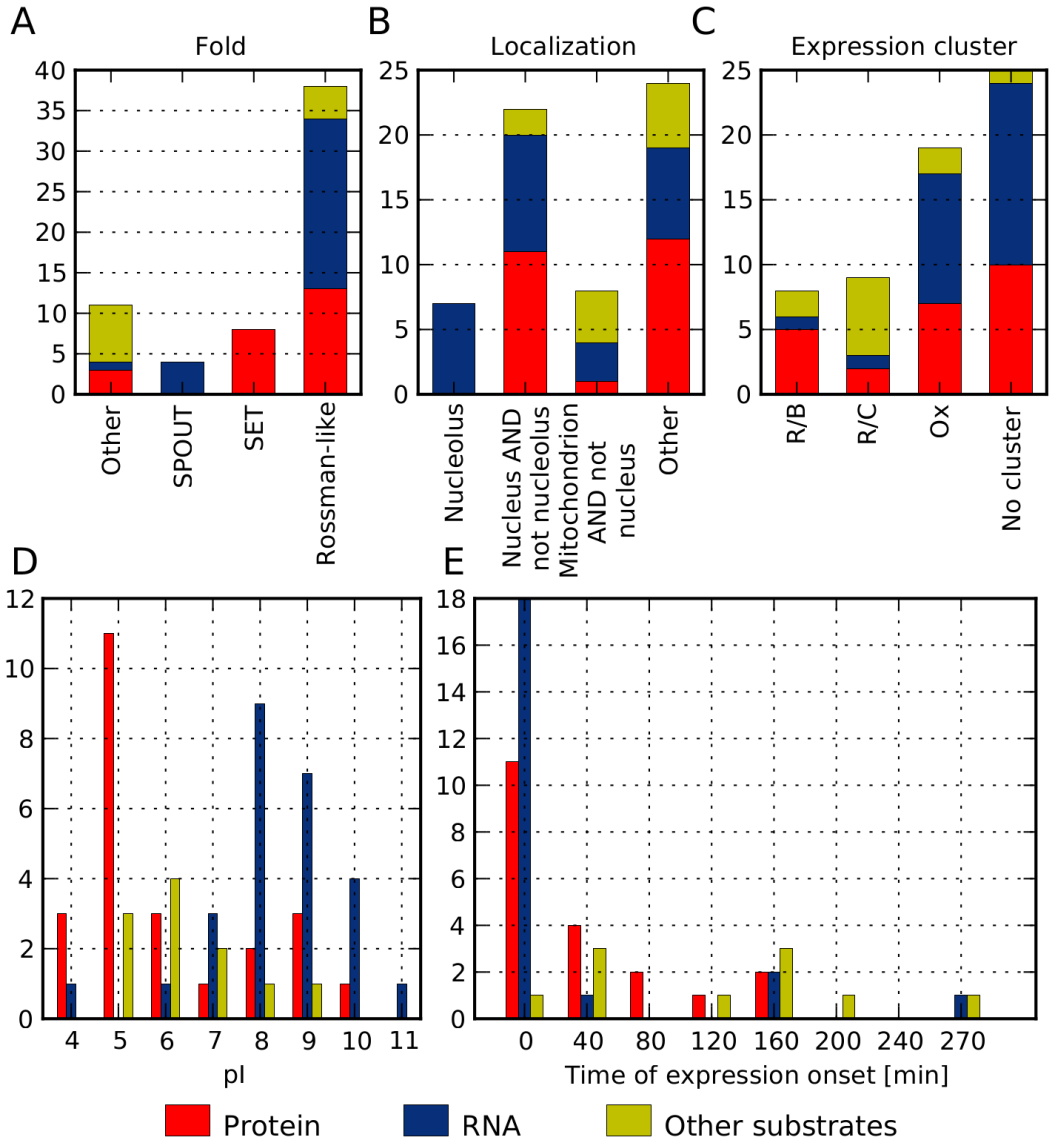
Distribution of various property classes among groups of MTases with different substrate specificity. (A) structural fold of the catalytic domain, (B) cellular localization, (C) expression cluster in YMC, (D) expression onset in the YMC, (E) isoelectric point (pI).

Preliminary selection of biophysical, cellular and functional properties of MTases to use in our model was based on our previous research [11], which indicated that protein isoelectric point (pI), structural fold, expression pattern, expression onset and cellular localization are all correlated with MTase substrate specificity (Fig. 1). We performed preliminary studies to determine which specific properties have the highest predictive power. Specifically, we interrogated similar properties to find out which among them are most significantly enriched in MTases sharing the same general substrate specificity. For example, we examined different data on protein cellular localization including both predicted localizations [17], which are available for all proteins, and experimentally derived data on protein localizations. We concluded that for yeast MTase substrate specificity predictions the most useful data are Gene Ontology protein localizations limited to IDA and IEA evidence codes and additionally grouped into superclusters of localizations. Similarly, we grouped structural folds into superclusters (Fig. 2A and B). The predictors selected for use in the models are discussed in detail below.

### Structural fold of MTase catalytic domain

As shown in our previous study [11], yeast MTases may adopt up to nine different folds (predicted with high confidence from sequence similarity) within their catalytic domains: Rossmann-like, SPOUT, SET domain, TIM beta/alpha-barrel, transmembrane, tetrapyrrole methylase, DNA/RNA-binding 3-helical bundle, SSo0622-like and thymidylate synthetase. For predictions, we divided them into four groups based on the frequency of a particular fold being assumed by MTases and correlation with their substrate specificity preference: Rossmann-like, SPOUT, SET domain and “other”. The “other folds” category was motivated by few yeast MTases assuming them and their shared preference for “other” substrate specificity (Fig. 2A). In contrast, all eight known MTases with a SET fold methylate proteins, and all four known MTases with a SPOUT fold methylate RNA (Fig. 2A). The Rossmann-like fold MTase group has more diverse substrate specificities and comprises 62% of known MTases. About two-thirds of MTases in the “other folds” category methylate other substrates.

### Cellular localization

We observed that for known MTases, substrate specificity correlates with cellular GO localizations [18], especially for the nucleolus, nucleus and mitochondrion localizations (Fig. 2B). Moreover, the original number of GO localization terms were clearly too big in comparison with the number of known MTases. Therefore, we decided to describe MTase cellular localization by four mutually exclusive terms: (i) nucleolus, (ii) nucleus *and* not nucleolus, (iii) mitochondrion *and* not nucleus, and (iv) other. All known yeast MTases localized in the nucleolus have RNA as a substrate. MTases with ‘nucleus *and* not in nucleolus’ localization most often methylate proteins (50%) or RNA (41%); only two methylate other substrates. Among known MTases within the ‘mitochondrion *and* not nucleus’ category there is only one example of a protein MTase. The remaining twenty three known protein MTases are not localized in the mitochondria. Moreover, MTases that methylate other substrates constitute 50% of those in the ‘mitochondrion *and* not nucleus’ group.

### Isoelectric point (pI)

As we pointed out in [11], for known MTases, global pI values correlate with their substrate specificity. Since the isoelectric point is a proxy for protein charge, we can expect proteins with a high pI to bind negatively charged molecules like RNA. Indeed, 67% of known MTases with pI≥6.5 methylate RNA. On the other hand, 65% of known MTases with a low pI<6.5 methylate proteins. MTases that methylate other substrates have a medium-range pI (Fig. 2D).

We also searched for regions with very high or low pI values, expecting that such regions of a protein might correspond to substrate binding regions or domains. For automatic identification of such regions, we computed the maximum and minimum local pI values for each sliding window size (from 15 to 185 a.a.) and for each MTase, and referred to them as pI max and pI min, respectively.

### Expression patterns in Yeast Metabolic Cycle (YMC)

The YMC is a redox cycle lasting 300 minutes, in which genes with similar functions tend to be expressed within a specific temporal window [19]. Expression profiles of genes periodically expressed in the YMC can be grouped into three main clusters: Ox (oxidative), R/C (reductive/charging) and R/B (reductive/building) [19]. Nineteen known MTases belong to the Ox cluster, among them ten methylate RNAs and seven methylate proteins. Two-thirds of known MTases from the R/C cluster methylate other substrates, and most of those from the R/B cluster (5 out of 8) methylate proteins (Fig. 2C). To describe expression patterns, in addition to YMC expression clusters, we also used the onset of individual YMC gene expression [20]. More than half of known MTases (30 of 52) have similar YMC expression onsets around the beginning of the YMC cycle (between 280 min and 16 min). However, all but one known MTase that methylate other substrates and have assigned the onset of expression [20], have expression onsets after 16 min and before 280 min (Fig. 2E) (only genes periodically regulated during YMC, as determined by [21] have their onsets of expression assigned).

### The best single-property models

The ML method was used to select a model most likely to reproduce the observed data (i.e. general substrate specificities of the known MTases) and then AIC penalty for number was parameters was applied. The model best scoring after the AIC correction is hereafter referred to as the “best” model. The properties, along with their parameterization, log likelihood and AIC values are listed in Table S1. Surprisingly, the best scoring single property is the isoelectric point (pI). The best model with a single pI threshold had a pI threshold of 6.97. The model using this single property can correctly predict substrate specificity for 67% of the known MTases, giving even better results than the single property model based on structural fold. The second best scoring property is pI max (calculated using 125 a.a. sliding window) with a threshold of 9.85. However, we do not expect the 125 a.a. to be a biophysically important fragment size, because when two thresholds for pI max are allowed, the best fragment size is much bigger (170 a.a.). The pI max model correctly predicts 42 out of 61 proteins (69%). The third best scoring property is the protein fold, single-property model using fold correctly predicts 66% of known MTases. (The models are ranked not according to the number of correct prediction, which is not a smooth measure and is subject to Poissonian noise, but according to their AIC value, which is log likelihood with the penalty for the number of parameters).

The prior probability of having a given substrate specificity, *P*(substrate*_i_*), that we used in our models was the fraction of known MTases with that specific substrate type. When we made predictions using prior probabilities alone, they were correct in only 43% of cases, while for the best single property model, they were correct in 67% of cases. We also verified that allowing a different *P*(substrate*_i_*) than that observed among known MTases does not improve the outcome: optimization over different prior probabilities converges to values observed among known MTases.

We compared our approach with a simple homology method of substrate specificity inference from a well annotated protein sharing the highest sequence similarity. Such prediction from the most similar known MTase of the same catalytic fold (the closest paralog) in *S. cerevisiae* gave 61% correct predictions for known MTases. This shows that in our case sequence similarity, contrary to popular belief, is not the most informative property for predicting MTase substrate specificity within a single organism, as even close homologs can have different general substrate specificities. For example, MTases PPM1 and PPM2 display ∼30% sequence identity, but methylate different types of substrates: PPM1 methylates a protein while PPM2 methylates an RNA.

### The best multi-property models

We studied predictive models using several properties at a time, assuming their independence (Eq. 2 Methods). In practice, the properties included are typically correlated. To address this issue, we first used the ML method to optimize parameter values for every family of the models considered (i.e. for any different combination of properties) to maximize the accuracy of predictions in known cases. Naturally, models with a higher number of parameters will produce more accurate predictions. Therefore, we used AIC for model selection to ensure that the model with the most informative properties, as opposed to the model using the most properties, would be chosen as our best model. Finally, such a chosen model (best model) was used to predict substrate specificities for 25 putative *S. cerevisiae* MTases with unknown substrate specificity (putative MTases).

We evaluated 86000 multi-property models dependent on up to 14 properties (Table S3 and S4). The best-scoring model uses the following properties: pI, SET fold, other folds and R/C expression cluster (Table S3). The pI property employs a single threshold of 6.95. Other properties, SET fold, other folds and R/C expression cluster, are binary properties; an MTase can either have this property or not. The pI property distinguishes known MTases that methylate RNA from those that methylate proteins, while the SET fold property indicates known MTases with protein substrate specificity. Analogously, the “other folds” property correlates with “other” substrate specificity. Detection of known MTases with other substrate specificity is additionally supported by including an R/C expression cluster category, which is employed by the top five models (Table S3). The sixth best model does not use any property derived from the expression data, but it does use localization (mitochondrion) and pI (with single threshold of 6.96), SET fold and “other folds” properties. The best model using neither localization or expression data utilizes pI (with single threshold of 6.97), SET fold and “other folds” properties. This model scores 35th in terms of best AIC and correctly predicts substrate specificities of 79% of known MTases (48 out of 61).

### Verification of the best model using known MTases

The best model correctly predicts substrate specificity for 83.6% of known MTases (in 51 out of 61 MTases the highest scoring substrate class coincided with the actual substrate class) (Table S5). We computed the statistical significance of obtaining 51 out of 61 correct MTase substrate specificity predictions with the null hypothesis that predictions are random. We then applied very conservative Bonferroni correction considering 86,000 alternative models for multiple hypothesis testing and obtained a very statistically significant *p*-value, *p* = 7.2×10^−9^, even though our search space for the best model was not restricted to the most promising candidates. This result shows that our method is capable of yielding final models with very high predictive power.

Moreover, the probabilities associated with the best scoring substrate specificity are significantly higher when the prediction is right than when it is not (*p* = 0.01, *t*-test, Fig. S1). Taken together, the overall very high-accuracy of our predictions (>83%) combined with the statistically significant correlation between correctness of our prediction and the likelihood we assign to predicted substrate specificities validates our approach and justifies the selection of classes of input parameters for our models (Fig. 1).

We succeeded in predicting substrate specificity for 88.5% (23 of 26) of RNA MTases, 70.8% (17 of 24) of protein MTases and 100% of 11 MTases that methylate other substrates. Among the MTases whose substrate specificity was not predicted correctly, four (YDL200C, YDR410C, YDR440W, YNL063W) were predicted to methylate RNA and three (YDR435C, YLR137W, YLR172C) to have other substrate specificity while they actually methylate proteins. For the last five of those MTases correct substrate specificity predictions have the second-highest probabilities. Namely, they are predicted to be protein MTases with the following probabilities: YLR172C (37%), YLR137W (36%), YDR435C (33%) and YDR440W and YNL063W (14%). Thus, known MTases methylating proteins appear to be the most difficult to predict, likely due to vast functional differences within the ‘protein’ class of substrates. On the other hand, we predicted three MTases (YDL112W, YOL141W, YOR239W) to have protein substrate specificity when in fact they are RNA MTases. Below we discuss in detail the reasons for incorrect predictions in these difficult cases: (i) ABP140 (YOR239W) has extraordinary low pI compared with other known MTases that methylate RNA; (ii) PPM1 (YDR435C) and PPM2 (YOL141W) are close homologs that methylate protein and RNA, respectively. However, they both modify the same chemical group: oxygen from a carbonyl group. Specifically, PPM1 methylates the C-terminal of protein phosphatase 2A [22], in turn PPM2 is involved in the methoxycarbonylation required for synthesis of wybutosine, an atypical nucleoside of tRNAPhe [23]. They have very similar pIs that are below our 6.95 threshold. Low pI is more typical for the known protein MTases, therefore PPM2 is predicted to methylate protein. Additionally, PPM1 is in the R/C expression cluster of the YMC, which outweighs its prediction towards methylating another substrate; (iii) MTQ1 (YNL063W), MGT1 (YDL200C), DOT1 (YDR440W), STE14 (YDR410C) are MTases that methylate proteins and are predicted to have RNA substrate specificity as they all have high pI (above 6.95 threshold). MGT1 is not a typical protein MTase because it transfers a methyl group from DNA to itself (DNA demethylation). The nucleic acid is not methylated, as predicted, but is actually a substrate in the reaction and the high positive charge of the MTase supports its binding. DOT1 is a Rossmann-like fold MTase specific for histones. We noticed a tendency for histone MTases to have relatively high pI (although it was not incorporated into our models due to there being only four histone MTases present in yeast). Specifically, SET1 and SET2 both methylate histones and also have a high pI, like DOT1 MTase. However, the model predicts them correctly as protein MTases because they have a SET fold. (iv) DPH5 (YLR172C) and YLR137W are protein MTases incorrectly predicted to methylate other substrate types. DPH5 has a tetrapyrrole methylase fold that is in the “other” folds category and YLR137W is in the R/C expression cluster. These properties overweigh prediction for those MTases to have other substrate specificity; (v) TRM3 (YDL112W) is an RNA MTase that is incorrectly predicted to methylate protein because of its low pI.

### Substrate predictions for MTases with unknown substrate specificity

**Figure 3:**
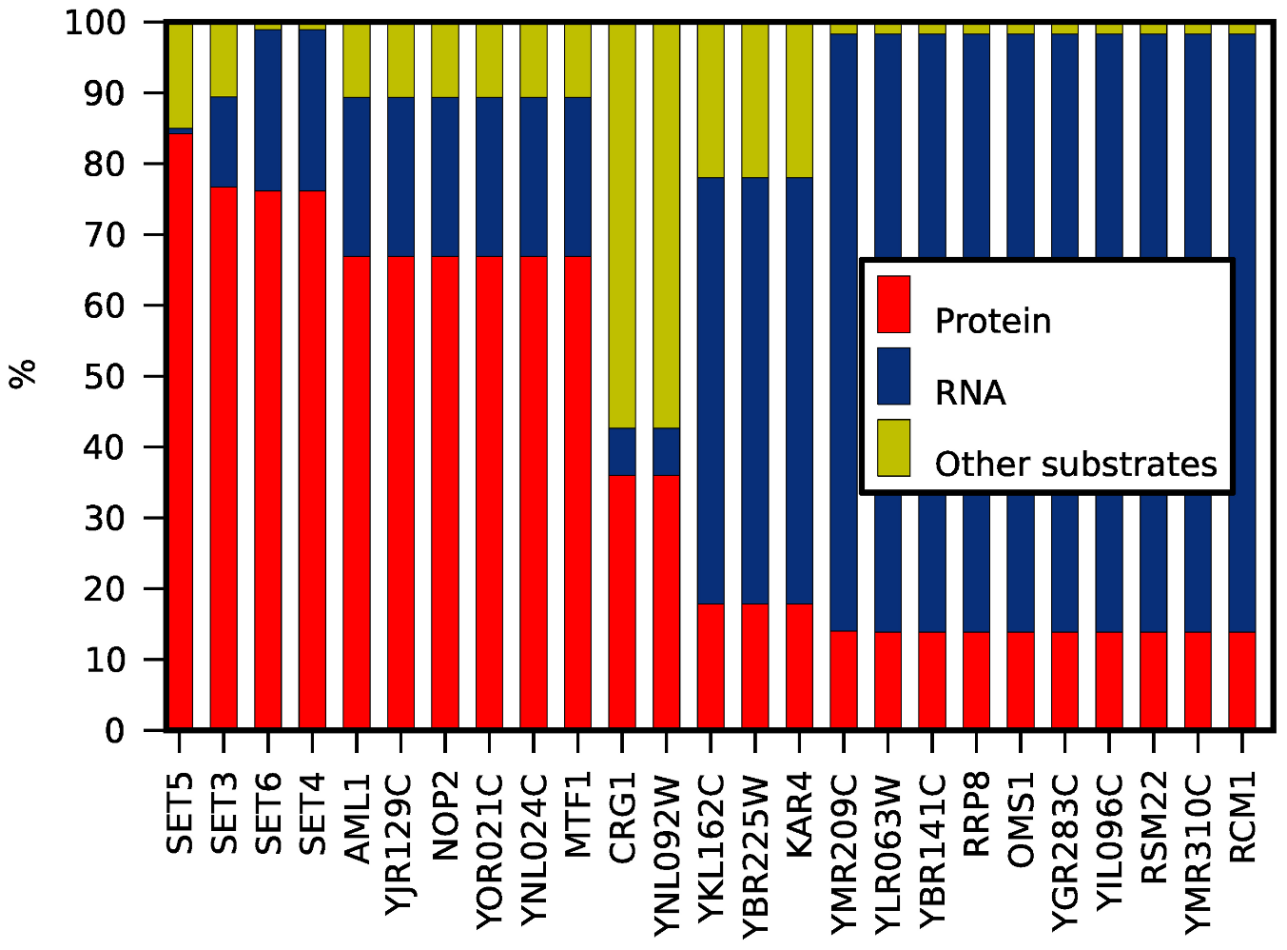
General substrate prediction for MTases with unknown substrate specificity.

According to our best model, 13 out of 25 putative MTases methylate RNAs, ten methylate proteins and two methylate other substrates (Fig. 3). Among 18 putative MTases with a Rossmann-like fold, five are predicted to methylate proteins, two to methylate other substrates and eleven to methylate RNA. As expected, all four putative MTases with a SET fold (YHR207C, YPL165C, YJL105W and YKR029C) are predicted to methylate proteins. Our model predicts two out the three putative MTases with a SPOUT fold (YGR283C, YMR310C) to methylate RNA. Surprisingly, our model also predicts that a third putative MTase with a SPOUT fold, YOR021C, is the first known example of a SPOUT methylase in any organism to methylate a substrate other than RNA [24].

### Experimental verification of substrate specificities predicted for putative MTases

To validate our approach for general substrate specificity prediction we performed protein methylation assays for selected putative yeast MTases. We used this approach successfully in the past to identify two yeast protein MTases: YBR271W and YLR285W (NNT1) [11]. Briefly, we incubated purified recombinant proteins with total cell extracts from the wild-type yeast and respective knockout strains in the presence of tritium-labeled AdoMet ([^3^H] AdoMet). The reaction products were analyzed by SDS-PAGE followed by autoradiography. HMT1 (a protein MTase) and TRM4 (an RNA MTase) were used as positive and negative controls, respectively. As expected, for control reactions we observed protein methylation patterns matching known substrates for HMT1, but not for RNA MTase TRM4 (the smear at the bottom of the gels in TRM4 lane corresponds to tRNA substrate).

**Figure 4:**
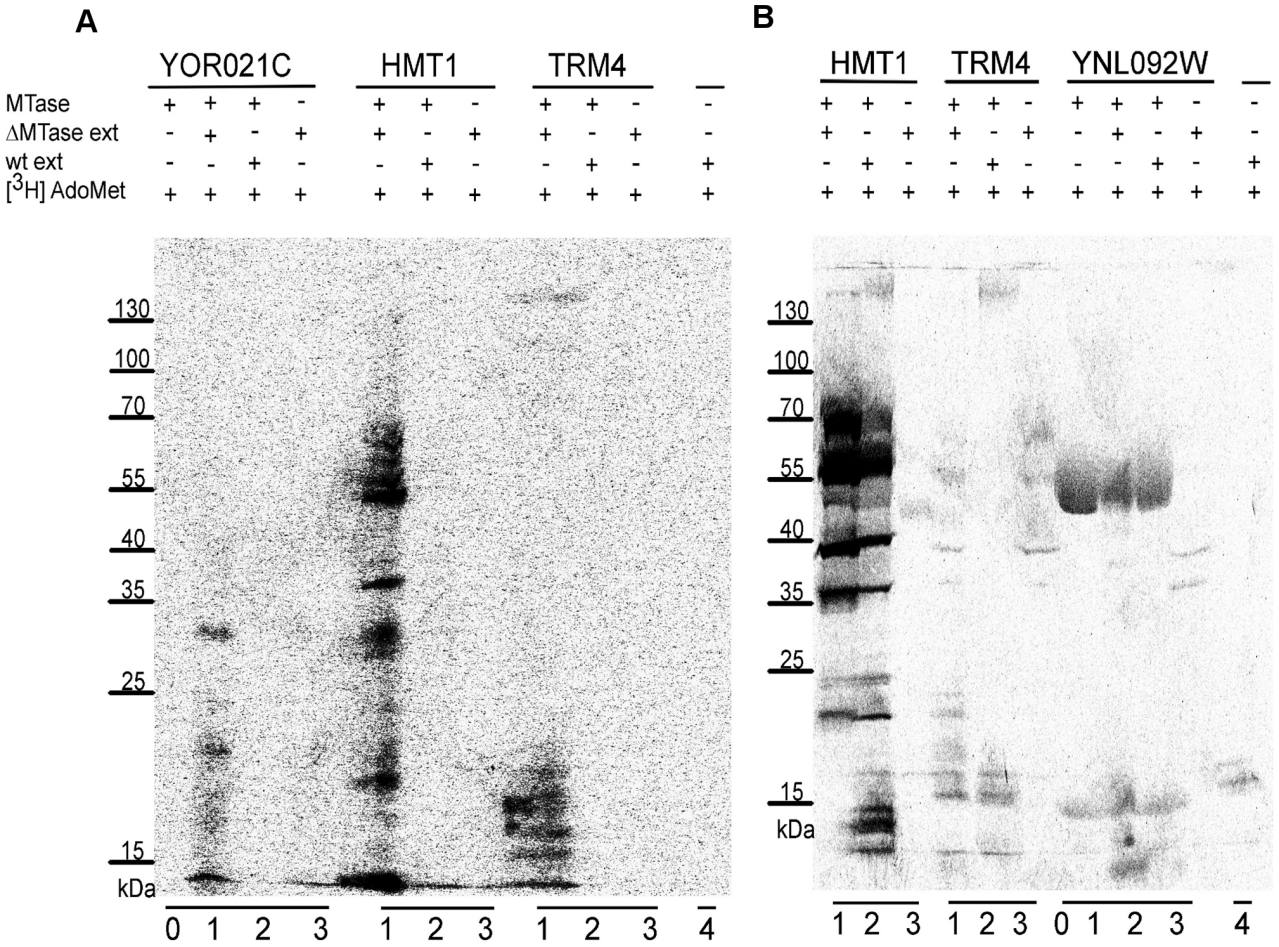
Experimental verification of substrate specificities predicted for putative MTases. (A) YOR021C and (B) YNL092W are protein MTases. Recombinant proteins (MTases) were incubated with native yeast extracts from the respective knockout strains (ΔMTase ext) and [^3^H] AdoMet (lane 1). Reaction products were resolved on SDS-PAGE gel and exposed to tritium screen. To test the specificity of the reactions, analyzed proteins were also incubated with yeast extract from the wild-type strain (wt ext) and [^3^H] AdoMet (lane 2). As a control, yeast extracts from knockout and wild-type strains were incubated with [^3^H] AdoMet only (lanes 3 and 4). In addition, selected proteins were also incubated with [^3^H] AdoMet only (lanes 0). HMT1 (a protein MTase) and TRM4 (an RNA MTase) were used as positive and negative controls, respectively.

First, we focused on our most unexpected prediction that YOR021C is the first ever known SPOUT MTase to methylate protein (Table S2). Indeed, in the *in vitro* assay, we observed the presence of protein methylation products for YOR021C. YOR021C seems to methylate at least 2 proteins (∼20 and 30 kDa) detected only when the deletion strain was used (Fig. 4), which strongly suggests that these modifications are specific and stable. The same results were obtained when total RNA was removed from cell extracts using RNaseA. Combined, these data indicate that YOR021C is a protein MTase. Very recently another group independently confirmed our findings by showing that this MTase methylates a small ribosomal subunit protein Rps3 [25], with molecular weight 26.5 kDa, consistent with one of our observed methylation products. In contrast, for SPOUT MTases YGR283C and YMR310C, which we predict to methylate their usual substrate, RNA, no protein methylation was found (Fig. S2).

We also tested protein methylation for selected Rossmann-like fold MTases with unknown substrate specificity: YNL092W, YDR316W (OMS1), YIL096C and YKL155C (RSM22). An *in vitro* MTase activity assay suggests that YNL092W is a protein MTase. For this MTase we detected on tritium screen a methylated product corresponding to the molecular weight of YNL092W. Moreover, methylated product was also detected when purified recombinant protein was incubated only with [^3^H] AdoMet (Fig. 4), indicating that YNL092W methylates itself (since no other protein substrate was present). Interestingly, this seems to be the second yeast protein, after MGT1, capable of automethylation. For the remaining Rossmann-like MTases: YDR316W (OMS1), YIL096C and YKL155C (RSM22), predicted to methylate RNA, we did not observe any protein methylation (Fig. S2), supporting their predicted substrate specificity.

Our prediction that YHR209W (CRG1) methylates substrates from the “other substrate” category, has been recently confirmed by Lissina *et al.* [26], who showed it methylates canthardin. Another of our predictions, that YHR207C (SET5) methylates protein, has also been recently confirmed showing it to methylate histone H4 [27].

### Comparison with CAFA predictors

In the year 2012 CAFA experiment, F-measure (a harmonic mean between precision and recall) was used to compare performance of different models [3]. The best scoring CAFA model (Jones-UCL group) achieved F-measure of 0.6 for predictions of molecular function, while our classifier has an F-measure of 0.84. The fact that our focused method performs so much better than the best general predictor is very reassuring, although not surprising. Constructing narrower predictors allows for selecting features most relevant to the properties being predicted, and if executed well, should result in much better predictions than from predictors aiming to predict more general molecular function categories.

### Potential further applications

The framework presented in this paper can be readily applied to other biological systems and questions. Below is a discussion of the most promising areas of application requiring only minor adaptations of the approach.

#### A. Inferring substrate specificity of MTases at a more detailed level

It would be of interest to predict also more detailed function of MTases, such as methylating histone and ribosomal proteins, DNA, rRNA, tRNA, other RNA, lipids, small molecules and other molecules. Unfortunately, there are too few yeast MTases to successfully train a classifier predicting more detailed substrate categories. For example, in the training set of 61 known yeast MTases there were only three histone MTases. On the other hand, there are more known MTases in human, for example there are already 26 known human MTases methylating histones [28], so we expect these more detailed predictions to be successful in the case of human proteins. Adapting our predictor to include more categories is rather straightforward, and can be achieved by either considering more probabilities (e.g. 10 instead of 3) as *n* in Eq.3; or by employing a hierarchical prediction method. In the latter case, in the first step, the same or similar general probabilities would be predicted (protein, RNA, other or protein, RNA, DNA, other), and in the next step finer prediction will be made within each top-level category, (for example, what are probabilities of a given MTase to methylate histone proteins, ribosomal proteins or other proteins, given that it is predicted to methylate protein).

#### B. Predicting different types of substrate classes for MTases

Modifying our framework to predict very different substrate categories (e.g. whether the methylated atom is sulfur, nitrogen, oxygen or carbon) is also technically straightforward. The probabilities of a given MTase methylating sulfur, nitrogen, oxygen or carbon atoms should be used instead of the probabilities of its methylating protein, RNA or other molecule. However, since this substrate classification according to the methylated atoms is very different from our protein/RNA/other classification, the input properties of the model should be selected *de novo*, by screening them for the correlation with the methylated atom, as described in the Materials and Methods section.

#### C. Modifying the model to predict substrate specificities of other enzymes

The presented mathematical framework is very flexible and can be used to predict the substrate of other classes of enzymes. An interesting application would be to infer substrate specificities for kinases from the FGGY family. Such kinases can have 9 different functions: L-ribulokinase, erythritol kinase, L-fuculokinase, glycerol kinase, gluconokinase, L-xylulose kinase, D-ribulokinase, Rhamnulo-kinase and xylulose kinase [8]. To predict these functions using our approach, one needs only to substitute “*substrate_i_*” with “*function_i_*” in formulas (1)–(3), using *n* = 9. As a training set, the set of 446 FGGY kinases annotated with high confidence in [8] should be used. As input model variables, data used successfully by Godzik and colleagues [8] should be used: sequence similarity, operons and regulons, known pathway and functional context, with or without supplementing with additional data sources.

#### D. Generalizing the model to predict GO categories

The proposed framework can also be used to infer GO categories, as in the CAFA experiment [3]. The primary fundamental difference stems from the fact that GO categories typically have substantial overlap, while in our approach the predicted properties are non-overlapping. To overcome this technical problem, the best solution appears to proceed as we did with localization data – to convert it semi-manually into exhaustive and disjoint categories. Specifically, we clustered GO localization terms for yeast MTases into four mutually exclusive terms: (i) nucleolus, (ii) nucleus *and* not nucleolus, (iii) mitochondrion *and* not nucleus, and (iv) other. The classification was motivated by researching correlation between different localizations and substrate specificity of MTases and also by the desire to balance the number of proteins in different categories. Clearly, for general GO function predictions hundreds of GO categories should be included, but grouping them, as explained above, into disjoint categories should be helpful. Another possibility to adapt the presented framework to predict general GO categories is to construct individual, independent predictors for each major GO category. That is a much more laborious solution, but should also yield more accurate results.

### General performance considerations

How many substrate categories can be successfully predicted is a difficult question to answer without specific knowledge of the system to be studied. It depends not only on the number of known examples, but also on the distribution of properties of interest. In our experience, the number of reliably predictable categories approximates the square root of the size of the training set. Clearly, predicting fewer classes yields a higher accuracy of inference. Moreover, it is also important to choose prediction categories such that they have comparable number of known examples and no single predicted category includes very few members. It is also highly desirable that variance within categories should be limited. In a given case, the feasible number of categories can be determined empirically, by verifying, as we did, if predictions are statistically significant as compared with random predictions. In the case of yeast MTases, they were highly significant for predicting general substrate specificity (protein, RNA, other), but as expected not significant for predicting more detailed substrate specificity (histone protein, ribosomal protein, other protein, rRNA, tRNA, other RNA, lipid, small molecule, other), where number of categories exceeds the square root of number of known examples, our rule of thumb for maximal number of predictable categories.

In summary, our predictions proved to be very accurate, yielding an 84% correct prediction rate when tested on a set of MTases with known substrate specificity. After our predictions were made, substrate specificities of 9 MTases were fully or partially confirmed experimentally by us or others [26], [27], [29], with results consistent with predictions in 89% (8 out of 9) of the cases. Our work also aids in understanding how observed general substrate specificities are achieved at the molecular level. For instance we show that, surprisingly, a global biophysical property, pI, impacts MTase substrate specificity more than structural fold. Likely, pI, which closely correlates with protein charge, retains such an impact on substrate specificity because it often determines whether an MTase will bind negatively charged molecules such as RNA, or typically positively charged protein substrates. We also show that knowledge of a substrate binding site or corresponding motifs, traditionally thought to be crucial, is not essential for highly accurate general substrate specificity predictions for yeast MTases.

Our models combine inference from many sources to estimate the probabilities of given MTases having various substrate specificities. Unlike previously used classification schemes [8], [10], [11], this approach allows us to predict substrate specificity not yet observed for a given class of MTases. Indeed, we made one such prediction: that YOR021C, a SPOUT fold MTase, methylates a protein. That prediction was very surprising, as all SPOUT MTases known to date, both in yeast and other organisms, exclusively methylate RNAs. Strikingly, at the time of publication of this paper, this prediction has been confirmed both by us and independently by another group in a newly published paper [29].

In summary, we have shown that our general probabilistic framework based on fundamental laws of probability and information theory is a powerful tool to predict substrate specificity of yeast MTases. Biological expertise is still very important in our approach, but it is used only to select the initial properties plausibly related to the intended prediction; otherwise the proposed approach is completely objective and self-learning. Moreover, our model can be easily updated with new knowledge by repeating the same calculations on the updated data set. To ensure that our work is broadly applicable, as input to our model we prioritized organism-independent properties, especially ones that can be derived from sequence data alone. Therefore, our approach is also applicable to MTases in other organisms and with modifications can be used to predict the substrate specificities of other enzymes, as we discussed in the examples given above. As in the recent CAFA experiment, we conclude that the best predictions are obtained from integration of varied data types. Accuracy of our predictions, as measured by F-measure employed by CAFA, is much better than that of the best CAFA predictor. This underscores our belief that a successful classifier designed to predict more narrow functional categories should always outperform more general predictors. Given that accuracy of protein function prediction is crucial for its usefulness, more focused predictions, of the type we present, will always be needed. In the future, most successful general function predictors may employ predictors like ours for predicting function subcategories.

## Materials and Methods

### Bayesian model

For each MTase, we calculate the probability that it has a given substrate specificity (e.g. RNA, protein or other molecule) based on its properties (Eq. 2):
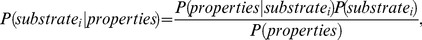
(2)For two different properties, for simplicity we assumed that they are independent. Specifically, the following equation was used:
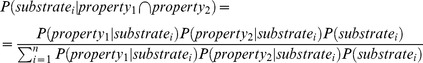
(3)where *n* is the number of substrate specificities.

*P*(property|substrate*_i_*) was calculated in different ways depending on whether the property is of the categorical or continuous type. (i) For categorical variables (e.g. localization, expression cluster), we estimated probabilities *P*(property|substrate*_i_*) for the whole population of MTases based on the sample of known MTases (Text S1. Supplementary text). (ii) For continuous variables (i.e. pI, expression onset), after dividing them into several intervals and estimating population values of *P*(property|substrate*_i_*) as in (i), we modeled them as a smoothed step function with two to three steps (specified by chosen thresholds) (Text S1. Supplementary text).

### Model selection

We tested 86,000 different combinations of up to 14 property types (Text S1. Supplementary text) by calculating likelihood of prediction for MTases with known substrate specificity. The best model was selected based on the lowest value of AIC, with AIC = 2k−2ln(L), where *k* is the number of parameters in the model and *L* is the maximized value of the likelihood function for the estimated model [16].

### Feature selection for modeling

Types of properties used in our model: structural fold, pI, expression pattern and cellular localization (Fig. 1 and Table S4), were selected based on our expert knowledge of which protein properties are relevant to MTase substrate specificity. Multiple properties belonging to these four broad categories were screened based on the statistical significance of their correlation with MTase substrate specificity. Supplementary table (Table S2) lists all *S. cerevisiae* MTases together with considered properties.

### Predictions based on sequence similarity

For comparison, we also predicted MTase substrate specificity using inference of substrate type from the closest paralog. Specifically, we assigned each yeast MTase a substrate specificity of an MTase with the same structural fold of catalytic domain and with the highest sequence similarity. To detect the closest yeast homolog, we used Meta-BASIC [30], a sensitive tool for recognition of distant similarity between proteins based on alignments of sequence profiles enriched with predicted secondary structure (meta profiles).

### Strains and media

The following yeast strains (Euroscarf) were used in this study: BY4741 (MATa his3Δ1 leu2Δ0 met15Δ0 ura3Δ0), BY4741 ΔYBL024W (ΔTRM4), BY4741 ΔYBR034C (ΔHMT1), BY4741 ΔYGR283C, BY4741 ΔYIL096C, BY4741 ΔYKL155C (ΔRSM22), BY4741 ΔYNL092W, BY4741 ΔYDR316W (ΔOMS1) BY4741 ΔYMR310C, BY4741 ΔYOR021C and BY4741 ΔYMR310C. The standard yeast genetic methods and selective growth media were used, as described in Rose et al. [31].

### Protein expression and purification

The following proteins: YBL024W (TRM4), YBR034C (HMT1), YGR283C, YIL096C, YKL155C (RSM22), YNL092W, YDR316W (OMS1), YOR021C and YMR310C were produced in *E. coli* (BL21-CodonPlus-RIL strain) as N-terminal HIStagSUMO tag fusions using LB medium and overnight IPTG inductions at 23°C. The bacterial pellets were lysed by sonication in buffer A (20 mM Tris-HCl pH 8.0, 200 mM NaCl, 10 mM imidazole, 10 mM 2-mercaptoethanol) and purified on His-Trap FF Crude columns (GE Healthcare). The proteins were further purified by size-exclusion chromatography on a Superdex 75 10/300 GL column (GE Healthcare) in buffer containing 10 mM Tris-HCl pH 8.0 and 150 mM NaCl. Finally, glycerol was added to the protein aliquotes (10% final concentration), which were then stored at −80°C. The purity and quantity of the proteins were assessed by SDS-PAGE.

### *In vitro* methylation assay

Yeast whole-cell extracts were prepared as previously described [32]. Recombinant proteins (5–15 µg) were incubated with 30 µg of native yeast extract (from wild-type and respective knockout strains) in the presence of [^3^H] AdoMet (0.5 µCi/reaction) in 20 µl of reaction buffer (10 mM HEPES pH 8.0, 2 mM EDTA, 50 mM KCl, 1 mM DTT). Reactions were incubated at room temperature for 1 hr, diluted 2-fold in Laemmli buffer and resolved on a 12% SDS-PAGE gel. The gel was stained with Coomassie blue, dried and exposed overnight to tritium screen.

## Supporting Information

Figure S1 **The average probabilities for MTases predicted correctly and incorrectly.** The average probabilities for MTases from the training set that were predicted correctly (left) are statistically significantly higher than for those predicted incorrectly (right). Boxes denote the average probabilities for dominant function specificity of an MTase for correct and incorrect predictions, respectively, error bars correspond to the variance of the mean.
(TIF)

Figure S2 **Experimental verification of substrate specificities.** Methylation assays for YGR283C, YMR310C, YDR316W, YIL096C and YKL155C. Recombinant proteins (MTase) were incubated with native yeast extracts from the respective knockout strains (ΔMTase ext) and [^3^H] AdoMet (lane 1). Reaction products were resolved on SDS-PAGE gel and exposed to tritium screen. To test the specificity of these reactions, analyzed proteins were also incubated with yeast extract from the wild-type strain (wt ext) and [^3^H] AdoMet (lane 2). As a control, yeast extracts from knockout and wild-type strains were incubated with [^3^H] AdoMet only (lanes 3 and 4). In addition, selected proteins were also incubated with [^3^H] AdoMet only (lanes 0). HMT1 (a protein MTase) and TRM4 (an RNA MTase) were used as positive and negative controls, respectively.
(TIF)

Figure S3
**Example of our smoothing of pI probability distribution.** We use the function: 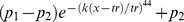 where *p_1_* = 0.75 and *p_2_* = 0.25 are average values of probability of assuming a given *pI* value within chosen intervals [4.16,6.95[ and [6.95,9.69] before smoothing, *tr* and *k* depend on the specific interval chosen, here *tr* = 4.17, *k* = 0.99.(TIF)

Figure S4
**Example of our smoothing of probability distribution of expression onset.** Note that since expression onset is a periodic variable in our case (the data comes from a periodic metabolic cycle, with period of 300 min), the probability density function is defined on a circle. Therefore, if only two intervals are considered, if plotted on a linear axis, it appears as three. We used the function: 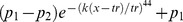, where *p_1_* = 0.22 and *p_2_* = 0.56 are average values of probability of assuming a given onset value within the chosen intervals [0,10[, [10,183[ and [183,300[ before smoothing; *tr* and *k* depend on the specific interval chosen, here *tr* = 86.5, *k* = 0.99.(TIF)

Table S1 **The models based on a single property.**
(DOC)

Table S2 **Properties of putative and known MTases used in the prediction model.**
(DOC)

Table S3 **The top 20 best models.**
(DOC)

Table S4 **Description of MTase properties tested in the model.** Beside properties described in the table, categorical property values were also used as independent properties with value true or false. Their names are: Ox, R/B, R/C, No cluster, Rossmann-like, SET, SPOUT, other fold, nucleus, nucleolus, mitochondrion, other localization. Those binary properties have 5 parameters.
(DOC)

Table S5 **Substrate specificity predictions for known MTases.**
(DOC)

Table S6 **Substrate specificity predictions for putative MTases.**
(DOC)

Text S1 **Supplementary text.**
(DOC)
