# Supplementary figures and images for: Probabilistic Approach to Predicting Substrate Specificity of Methyltransferases
Source: PLoS Comput Biol. 2014 Mar 20;10(3):e1003514. doi: 10.1371/journal.pcbi.1003514 (PMC3961171; doi:10.1371/journal.pcbi.1003514)

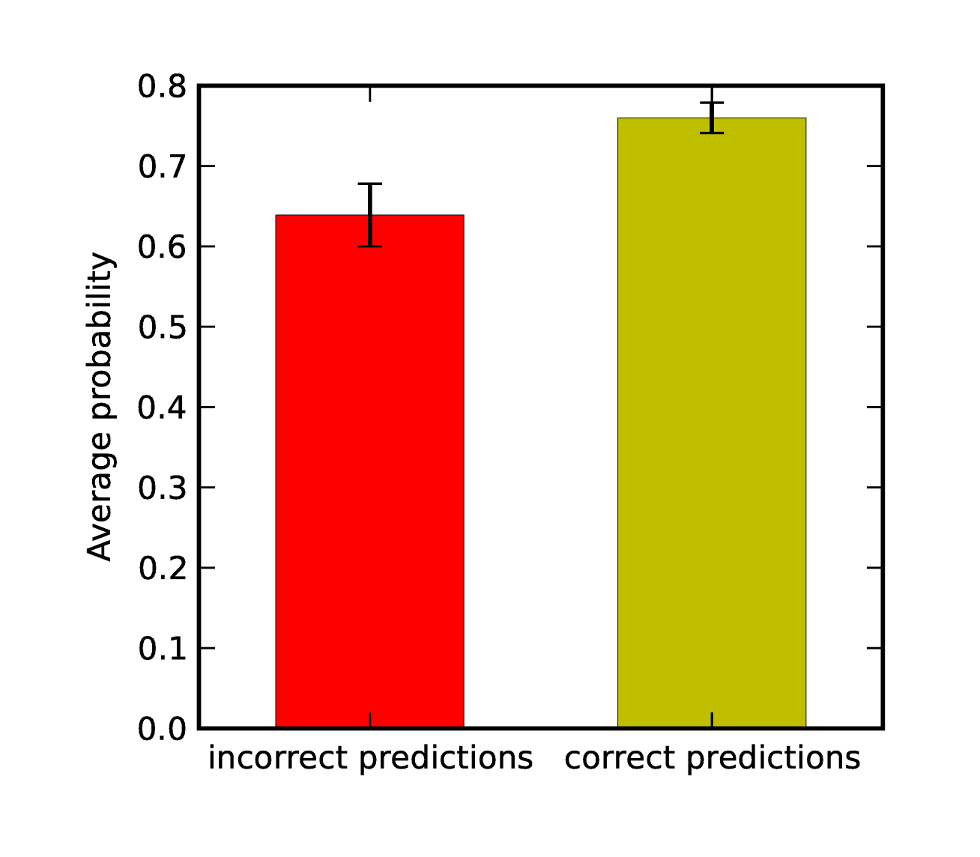

Supplement: Figure S1 — The average probabilities for MTases predicted correctly and incorrectly. The average probabilities for MTases from the training set that were predicted correctly (left) are statistically significantly higher than for those predicted incorrectly (right). Boxes denote the average probabilities for dominant function specificity of an MTase for correct and incorrect predictions, respectively, error bars correspond to the variance of the mean. (TIF) [file pcbi.1003514.s001.tif]

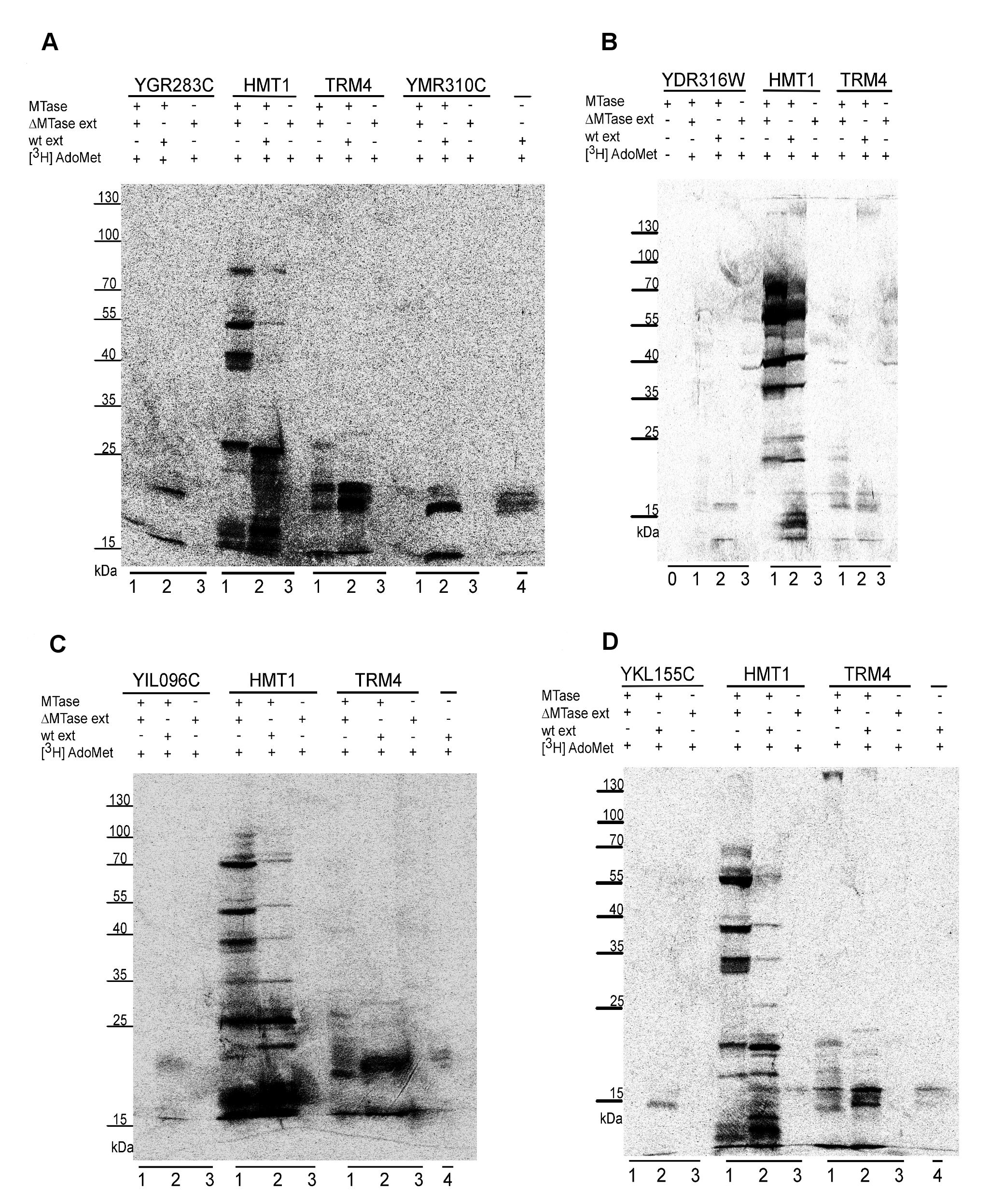

Supplement: Figure S2 — Experimental verification of substrate specificities. Methylation assays for YGR283C, YMR310C, YDR316W, YIL096C and YKL155C. Recombinant proteins (MTase) were incubated with native yeast extracts from the respective knockout strains (ΔMTase ext) and [3H] AdoMet (lane 1). Reaction products were resolved on SDS-PAGE gel and exposed to tritium screen. To test the specificity of these reactions, analyzed proteins were also incubated with yeast extract from the wild-type strain (wt ext) and [3H] AdoMet (lane 2). As a control, yeast extracts from knockout and wild-type strains were incubated with [3H] AdoMet only (lanes 3 and 4). In addition, selected proteins were also incubated with [3H] AdoMet only (lanes 0). HMT1 (a protein MTase) and TRM4 (an RNA MTase) were used as positive and negative controls, respectively. (TIF) [file pcbi.1003514.s002.tif]

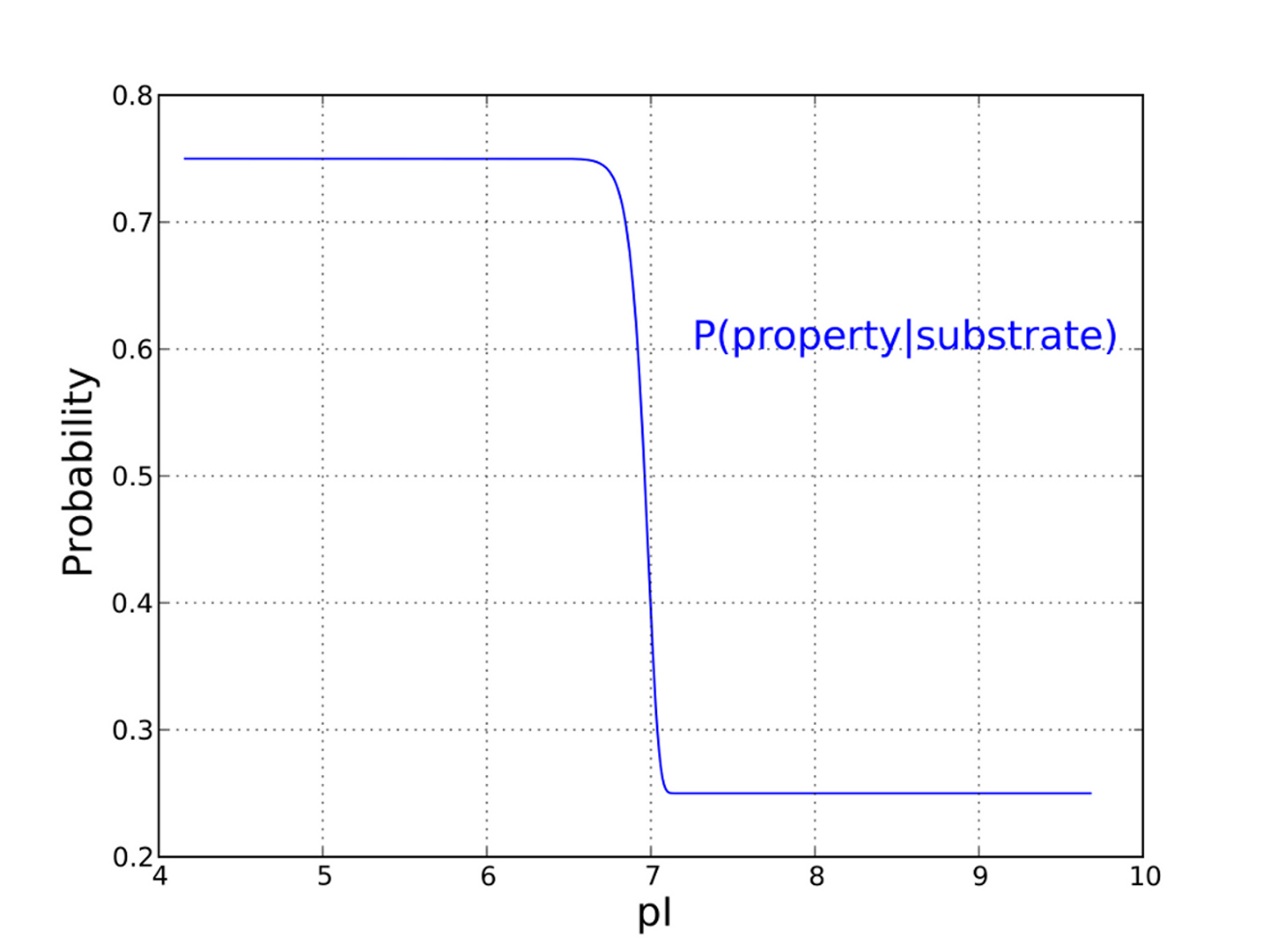

Supplement: Figure S3 — Example of our smoothing of pI probability distribution. We use the function: where p1 = 0.75 and p2 = 0.25 are average values of probability of assuming a given pI value within chosen intervals [4.16,6.95[ and [6.95,9.69] before smoothing, tr and k depend on the specific interval chosen, here tr = 4.17, k = 0.99. (TIF) [file pcbi.1003514.s003.tif]

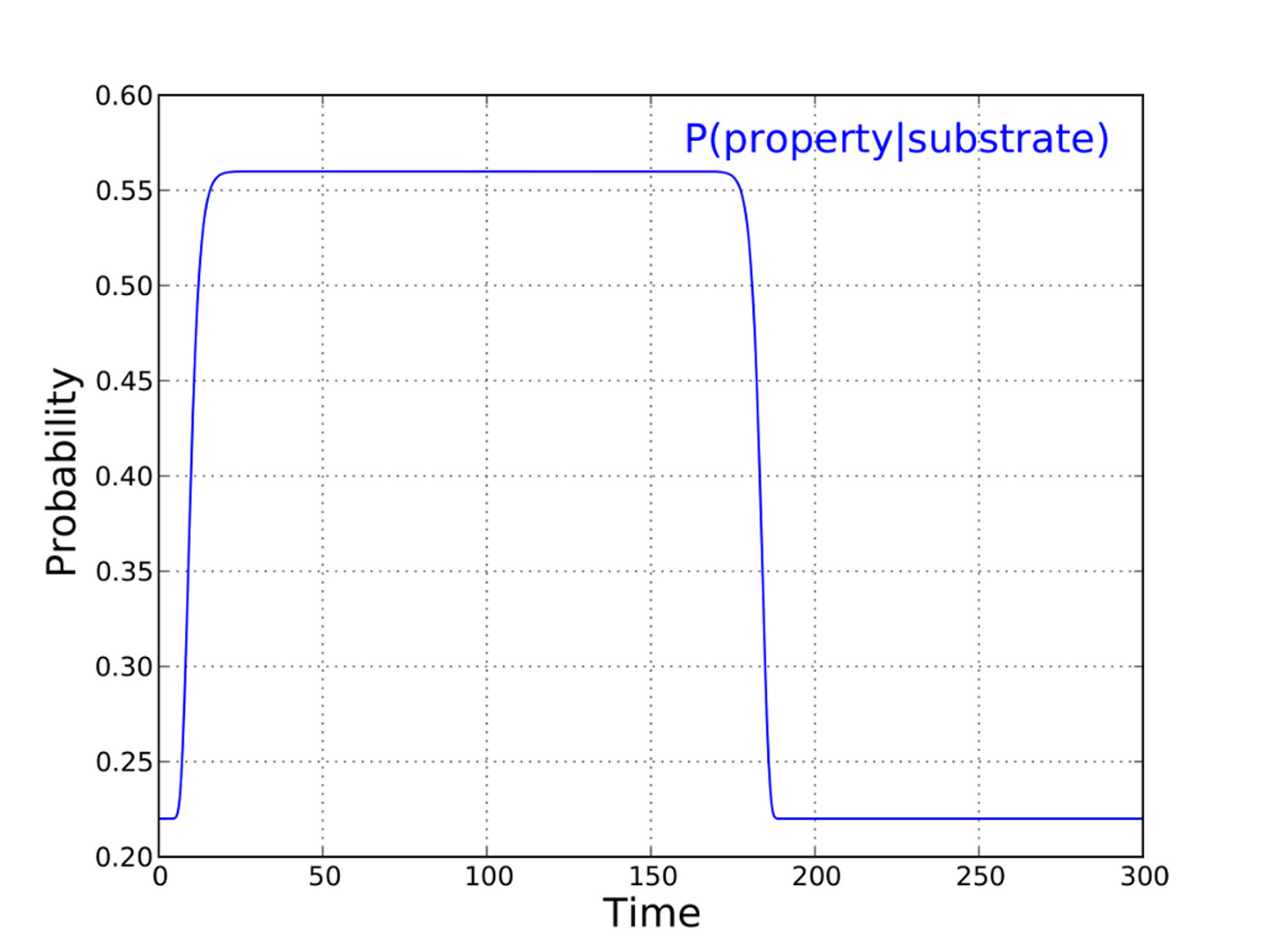

Supplement: Figure S4 — Example of our smoothing of probability distribution of expression onset. Note that since expression onset is a periodic variable in our case (the data comes from a periodic metabolic cycle, with period of 300 min), the probability density function is defined on a circle. Therefore, if only two intervals are considered, if plotted on a linear axis, it appears as three. We used the function: , where p1 = 0.22 and p2 = 0.56 are average values of probability of assuming a given onset value within the chosen intervals [0,10[, [10,183[ and [183,300[ before smoothing; tr and k depend on the specific interval chosen, here tr = 86.5, k = 0.99. (TIF) [file pcbi.1003514.s004.tif]
