# Supplementary material for: Probabilistic Approach to Predicting Substrate Specificity of Methyltransferases
Source: PLoS Comput Biol. 2014 Mar 20;10(3):e1003514. doi: 10.1371/journal.pcbi.1003514 (PMC3961171; doi:10.1371/journal.pcbi.1003514)
Supplement: Table S1 — The models based on a single property. (DOC) [file pcbi.1003514.s005.doc]

**Table S1. The models based on single property.**

| - Ln(likelihood) | Number of parameters | AIC | Property |
| --- | --- | --- | --- |
| 49.99 | 7 | 113.98 | pI (6.97) |
| 49.63 | 8 | 115.26 | pI max (9.85) 125 a.a. |
| 47.12 | 11 | 116.23 | Fold |
| 47.68 | 11 | 117.36 | pI max (7.98, 9.65) 170 a.a. |
| 48.73 | 10 | 117.47 | pI (6.89, 8.26) |
| 53.83 | 7 | 121.67 | Expression onset (9, 164) |
| 55.24 | 6 | 122.48 | Other folds |
| 56.12 | 6 | 124.24 | SET fold |
| 51.51 | 11 | 125.03 | Expression onset (8, 69, 272) |
| 56.71 | 6 | 125.42 | R/C expression cluster |
| 57.93 | 6 | 127.86 | Nucleolus |
| 53.53 | 11 | 129.07 | pI min (4.14, 4.36) 155 a.a. |
| 53.56 | 11 | 129.11 | Expression clusters |
| 56.56 | 8 | 129.12 | pI min (4.44) 185 a.a. |
| 54.25 | 11 | 130.50 | Localization |
| 59.49 | 6 | 130.99 | Rossmann-like fold |
| 59.81 | 6 | 131.61 | No cluster |
| 60.32 | 6 | 132.65 | Nucleus |
| 63.40 | 3 | 132.81 | No (only prior probability) |
| 60.49 | 6 | 132.97 | Mitochondrion |
| 60.80 | 6 | 133.61 | SPOUT |
| 61.51 | 6 | 135.02 | R/B expression cluster |
| 62.62 | 6 | 137.23 | Ox expression cluster |
