# Supplementary material for: Probabilistic Approach to Predicting Substrate Specificity of Methyltransferases
Source: PLoS Comput Biol. 2014 Mar 20;10(3):e1003514. doi: 10.1371/journal.pcbi.1003514 (PMC3961171; doi:10.1371/journal.pcbi.1003514)
Supplement: Table S2 — Properties of putative and known MTases used in the prediction model. (DOC) [file pcbi.1003514.s006.doc]

**Table S2**. Properties of putative and known MTases used in the prediction model.

| Systematic Name | Standard Name | Substrate | Substrate literature | Substrate specificity | pI | Fold | Expression cluster | Localization | Time [min] | Periodicity score |
| --- | --- | --- | --- | --- | --- | --- | --- | --- | --- | --- |
| YBL024W | TRM4 | tRNA | PMID: 10445884 | RNA | 6.97 | Rossmann-like | No cluster | Nucleolus | 15 | 1.39 |
| YBR030W | RKM3 | Rpl42ab | PMID: 18957409 | Protein | 4.27 | SET | No cluster | Nucleus | 298 | 1.47 |
| YBR034C | HMT1 | Npl3 | PMID: 8668183 | Protein | 5.09 | Rossmann-like | Ox | Nucleus | 296 | 2.88 |
|  |  | Hrp1 | PMID: 15314027 |  |  |  |  |  |  |  |
|  |  | Nab2 | PMID: 11779864 |  |  |  |  |  |  |  |
|  |  | Yra1 | PMID: 15314027 |  |  |  |  |  |  |  |
|  |  | Gar1 | PMID: 12756332 |  |  |  |  |  |  |  |
|  |  | Nop1 | PMID: 12756332 |  |  |  |  |  |  |  |
|  |  | Nsr1 | PMID: 12756332 |  |  |  |  |  |  |  |
|  |  | Rps2 | PMID: 20035717 |  |  |  |  |  |  |  |
| YBR061C | TRM7 | tRNA | PMID: 11927565 | RNA | 7.63 | Rossmann-like | No cluster | - | - | 1.07 |
| YBR133C | HSL7 | Calf thymus histone H2A | PMID: 10903903 | Protein | 5.89 | Rossmann-like | R/B | - | 96 | 3.68 |
| YBR141C | YBR141C | - | - | - | 10.06 | Rossmann-like | Ox | Nucleolus | 288 | 2.59 |
| YBR225W | YBR225W | - | - | - | 8.0 | Rossmann-like | R/C | - | 166 | 3.86 |
| YBR236C | ABD1 | mRNA | PMID: 7623811 | RNA | 7.81 | Rossmann-like | No cluster | Nucleus | - | -0.58 |
| YBR261C | TAE1 | Rpl12ab Rps25a/Rps25b | PMID: 20481588 | Protein | 4.81 | Rossmann-like | Ox | - | 12 | 2.3 |
| YBR271W | YBR271W | Uncharacterized proteins | PMID: 21858014 | Protein | 4.63 | Rossmann-like | No cluster | - | 290 | 1.86 |
| YCL054W | SPB1 | 60S rRNA | PMID: 14636587 | RNA | 8.0 | Rossmann-like | No cluster | Nucleolus | 1 | 1.29 |
| YCL055W | KAR4 | - | - | - | 8.38 | Rossmann-like | R/C | Nucleus | 169 | 3.7 |
| YCR047C | BUD23 | 18S rRNA | PMID: 18332120 | RNA | 9.31 | Rossmann-like | No cluster | Nucleolus | 5 | 1.76 |
| YDL014W | NOP1 | Pre-rRNA | PMID: 8431947 | RNA | 10.63 | Rossmann-like | Ox | Nucleolus | 5 | 2.18 |
| YDL112W | TRM3 | tRNA | PMID: 9917067 | RNA | 5.66 | SPOUT | No cluster | - | 7 | 1.53 |
| YDL200C | MGT1 | MGT1 | PMID: 19164530 | Protein | 8.75 | Other folds | No cluster | Nucleus | - | 1.64 |
| YDL201W | TRM8 | Pre-tRNA Phe | PMID: 12403464 | RNA | 9.41 | Rossmann-like | No cluster | Nucleus | 6 | 1.76 |
| YDR083W | RRP8 | - | - | - | 9.88 | Rossmann-like | No cluster | Nucleolus | 2 | 1.69 |
| YDR120C | TRM1 | Mitochondrial and cytoplasmic tRNA | PMID: 2426253 | RNA | 9.31 | Rossmann-like | No cluster | Nucleus, mitochondrion | 0 | 1.67 |
| YDR140W | MTQ2 | Sup45p(eRF1) | PMID: 16321977 | Protein | 4.72 | Rossmann-like | No cluster | Nucleus | 8 | 1.34 |
| YDR198C | RKM2 | Rpl12ab | PMID: 17005568 | Protein | 5.0 | SET | No cluster | - | 283 | 1.37 |
| YDR257C | RKM4 | Rpl42ab | PMID: 18957409 | Protein | 4.74 | SET | No cluster | Nucleus | 12 | 1.55 |
| YDR316W | OMS1 | - | - | - | 9.78 | Rossmann-like | R/B | Mitochondrion | 48 | 4.39 |
| YDR410C | STE14 | a-factor (MFA) | PMID: 2050108 | Protein | 7.63 | Other folds | Ox | - | 26 | 2.02 |
|  |  | RAS1 |  |  |  |  |  |  |  |  |
|  |  | RAS2 |  |  |  |  |  |  |  |  |
| YDR435C | PPM1 | PP2A | PMID: 11697862 | Protein | 6.88 | Rossmann-like | R/C | - | 154 | 2.8 |
| YDR440W | DOT1 | H3 | PMID:15292170 | Protein | 9.31 | Rossmann-like | R/B | Nucleus | 44 | 3.71 |
| YDR465C | RMT2 | Rpl12ab | PMID:17005568 | Protein | 4.16 | Rossmann-like | Ox | Nucleus | 298 | 2.68 |
| YER091C | MET6 | Homocysteine | PMID: 16083849 | Other substrates | 6.03 | Other folds | Ox | - | 20 | 4.22 |
| YER175C | TMT1 | Trans-aconitate | PMID: 11695919 | Other substrates | 5.75 | Rossmann-like | R/C | - | 260 | 3.66 |
| YGL050W | TYW3 | tRNAPhe | PMID: 16642040 | RNA | 7.81 | Other folds | No cluster | - | - | 1.11 |
| YGL136C | MRM2 | 21S rRNA | PMID: 11867542 | RNA | 9.13 | Rossmann-like | No cluster | Mitochondrion | - | 0.85 |
| YGL192W | IME4 | mRNA | PMID: 20421205 | RNA | 7.81 | Rossmann-like | R/C | Nucleus | 172 | 3.44 |
| YGR001C | AML1 | - | - | - | 4.53 | Rossmann-like | No cluster | - | 248 | 2.06 |
| YGR157W | CHO2 | Phosphatidyl-ethanolamine | PMID: 2445736 | Other substrates | 8.28 | Other folds | R/C | - | 197 | 3.34 |
| YGR283C | YGR283C | - | - | - | 9.59 | SPOUT | No cluster | Nucleolus | 4 | 1.44 |
| YHL039W | EFM1 | eEF1A | PMID: 20510667 | Protein | 6.13 | SET | No cluster | - | - | 1.1 |
| YHR070W | TRM5 | tRNA | PMID: 11226173 | RNA | 8.94 | Rossmann-like | Ox | Nucleus | 296 | 2.56 |
| YHR109W | CTM1 | iso-1-cytochrome c | PMID: 10791961 | Protein | 4.63 | SET | R/B | - | 52 | 3.88 |
| YHR119W | SET1 | H3 | PMID: 11805083 | Protein | 9.22 | SET | No cluster | Nucleus | - | 0.26 |
|  |  | Dam1 | PMID: 16143104 |  |  |  |  |  |  |  |
| YHR207C | SET5 | - | - | - | 6.03 | SET | R/C | Nucleus | 146 | 2.29 |
| YHR209W | CRG1 | - | - | - | 5.56 | Rossmann-like | R/C | - | 152 | 4.03 |
| YIL064W | SEE1 | eEF1A | PMID: 20510667 | Protein | 4.72 | Rossmann-like | Ox | - | 16 | 2.34 |
| YIL096C | YIL096C | - | - | - | 10.11 | Rossmann-like | No cluster | Nucleolus | 282 | 1.58 |
| YIL110W | HPM1 | Rpl3 | PMID: 20864530 | Protein | 4.27 | Rossmann-like | No cluster | Nucleus | 10 | 1.91 |
| YJL105W | SET4 | - | - | - | 8.56 | SET | No cluster | - | - | -0.2 |
| YJL125C | GCD14 | tRNA | PMID: 10779558 | RNA | 7.25 | Rossmann-like | Ox | Nucleus | 299 | 2.02 |
| YJL168C | SET2 | H3 | PMID: 12736296 | Protein | 8.38 | SET | R/B | Nucleus | 60 | 3.62 |
| YJR073C | OPI3 | Methylene-fatty-acyl-phospholipid | PMID: 2445736 | Other substrates | 9.13 | Other folds | R/C | Mitochondrion | 121 | 4.22 |
| YJR129C | YJR129C | - | - | - | 4.72 | Rossmann-like | Ox | - | 300 | 2.38 |
| YKL155C | RSM22 | - | - | - | 9.78 | Rossmann-like | R/B | Mitochondrion | 74 | 4.03 |
| YKL162C | YKL162C | - | - | - | 7.63 | Rossmann-like | R/C | Mitochondrion | 166 | 3.59 |
| YKR029C | SET3 | - | - | - | 8.84 | SET | R/C | - | 23 | 2.13 |
| YKR056W | TRM2 | tRNA | PMID: 10864043 | RNA | 8.94 | Rossmann-like | Ox | - | 280 | 2.46 |
| YKR069W | MET1 | Uroporphyrino-gen III | PMID: 10051442 | Other substrates | 6.13 | Other folds | Ox | - | 19 | 3.42 |
| YLL062C | MHT1 | Homocysteine | PMID: 17264075 | Other substrates | 5.19 | Other folds | R/B | - | 36 | 3.42 |
| YLR063W | YLR063W | - | - | - | 7.63 | Rossmann-like | Ox | - | 2 | 3.03 |
| YLR137W | YLR137W | Rpl1ab | PMID: 21460220 | Protein | 5.84 | Rossmann-like | R/C | - | 158 | 2.88 |
| YLR172C | DPH5 | EEF2 | PMID: 16950777 | Protein | 4.58 | Other folds | Ox | - | 14 | 2.7 |
| YLR186W | EMG1 | 18S rRNA | PMID: 20972225 | RNA | 8.19 | SPOUT | No cluster | Nucleolus | 8 | 1.74 |
| YLR285W | NNT1 | Uncharacterized protein | PMID: 21858014 | Protein | 4.81 | Rossmann-like | Ox | - | 115 | 2.18 |
| YML008C | ERG6 | 5alpha-cholesta-8,24-dien-3beta-ol | PMID: 2677674 | Other substrates | 5.38 | Rossmann-like | R/C | Mitochondrion | 158 | 3.39 |
| YML014W | TRM9 | tRNAArg3; tRNAGlu | PMID: 14645538 | RNA | 8.19 | Rossmann-like | Ox | Nucleus | 4 | 2.35 |
| YML110C | COQ5 | 2-hexaprenyl-6-methoxy-1,4-benzoquinone | PMID: 9083048 | Other substrates | 6.13 | Rossmann-like | R/C | Mitochondrion | 144 | 3.06 |
| YMR209C | YMR209C | - | - | - | 7.25 | Rossmann-like | No cluster | - | - | 0.78 |
| YMR228W | MTF1 | - | - | - | 6.13 | Rossmann-like | R/B | Mitochondrion | 84 | 3.99 |
| YMR310C | YMR310C | - | - | - | 9.88 | SPOUT | Ox | Nucleus | 6 | 2.28 |
| YNL022C | RCM1 | - | - | - | 8.75 | Rossmann-like | No cluster | Nucleus | 2 | 1.45 |
| YNL024C | YNL024C | - | - | - | 5.75 | Rossmann-like | No cluster | - | 2 | 1.49 |
| YNL061W | NOP2 | - | - | - | 4.72 | Rossmann-like | Ox | Nucleolus | 6 | 2.43 |
| YNL063W | MTQ1 | Mrf1 | PMID: 16321977 | Protein | 9.69 | Rossmann-like | No cluster | Mitochondrion | - | -0.28 |
| YNL092W | YNL092W | - | - | - | 5.56 | Rossmann-like | R/C | - | 233 | 3.51 |
| YOL093W | TRM10 | tRNA | PMID: 12702816 | RNA | 9.59 | SPOUT | Ox | Nucleus | 6 | 2.57 |
| YOL096C | COQ3 | 3,4-dihydroxy-5-all-trans-polyprenyl-benzoate | PMID: 10419476 | Other substrates | 6.5 | Rossmann-like | R/C | Mitochondrion | 142 | 2.98 |
| YOL124C | TRM11 | tRNA | PMID: 15899842 | RNA | 7.63 | Rossmann-like | Ox | - | 293 | 2.26 |
| YOL125W | TRM13 | tRNA | PMID: 17242307 | RNA | 8.09 | Rossmann-like | Ox | Nucleus | 300 | 2.25 |
| YOL141W | PPM2 | tRNAPhe | PMID: 19287006 | RNA | 6.59 | Rossmann-like | No cluster | Mitochondrion | 6 | 1.29 |
| YOR021C | YOR021C | - | - | - | 4.67 | SPOUT | No cluster | - | - | 1.54 |
| YOR074C | CDC21 | 5,10-methylene-tetrahydrofolate | PMID: 334734 | Other substrates | 6.88 | Other folds | R/B | Nucleus | 46 | 4.34 |
| YOR201C | MRM1 | 21S rRNA | PMID: 8266080 | RNA | 9.5 | SPOUT | R/B | Mitochondrion | 42 | 4.44 |
| YOR239W | ABP140 | tRNA | PMID: 21518804 | RNA | 4.37 | Rossmann-like | Ox | - | 16 | 3.16 |
| YPL030W | TRM44 | tRNASer | PMID: 18025252 | RNA | 8.56 | Rossmann-like | No cluster | - | 293 | 1.78 |
| YPL157W | TGS1 | snRNA, snoRNA | PMID: 11983179 | RNA | 9.83 | Rossmann-like | Ox | Nucleolus | 170 | 2.4 |
| YPL165C | SET6 | - | - | - | 7.44 | SET | No cluster | - | - | 2.79 |
| YPL208W | RKM1 | Rpl23ab | PMID: 16096273 | Protein | 4.77 | SET | R/B | Nucleus | 49 | 2.01 |
| YPL266W | DIM1 | 18S rRNA | PMID: 8064863 | RNA | 9.97 | Rossmann-like | No cluster | Nucleolus | 2 | 1.78 |
| YPL273W | SAM4 | Homocysteine | PMID: 11013242 | Other substrates | 4.91 | Other folds | No cluster | Nucleus | - | 1.22 |
