# Supplementary material for: Probabilistic Approach to Predicting Substrate Specificity of Methyltransferases
Source: PLoS Comput Biol. 2014 Mar 20;10(3):e1003514. doi: 10.1371/journal.pcbi.1003514 (PMC3961171; doi:10.1371/journal.pcbi.1003514)
Supplement: Table S3 — The top 20 best models. (DOC) [file pcbi.1003514.s007.doc]

Table S3. The top 20 best models.

| -Ln(likelihood) | Number of parameters | AIC | Properties | | | | | |
| --- | --- | --- | --- | --- | --- | --- | --- | --- |
| 29.31 | 15 | 88.61 | pI (6.95) |  |  | SET fold | Other folds | R/C |
| 24.50 | 21 | 91.00 | pI max (9.12) 185 a.a | Time (9,164) |  | SET fold | Other folds | R/C |
| 27.68 | 18 | 91.36 | pI (6.53) |  | SPOUT fold | SET fold | Other folds | R/C |
| 27.90 | 18 | 91.81 | pI (6.57) |  | R/B | SET fold | Other folds | R/C |
| 28.03 | 18 | 92.06 | pI (7.01) |  | Nucleolus | SET fold | Other folds | R/C |
| 28.06 | 18 | 92.12 | pI (6.96) |  | Mitochondrion | SET fold | Other folds |  |
| 30.11 | 16 | 92.21 | pI max (9.50) 110 a.a. |  |  | SET fold | Other folds | R/C |
| 30.11 | 16 | 92.22 | pI max (9.88) 125 a.a |  |  | SET fold | Other folds | R/C |
| 34.21 | 12 | 92.42 | pI (6.52) |  |  |  | Other folds | R/C |
| 30.25 | 16 | 92.50 | pI max (8.75) 185 a.a |  |  | SET fold | Other folds | R/C |
| 30.27 | 16 | 92.53 | pI max (9.74) 140 a.a |  |  | SET fold | Other folds | R/C |
| 25.28 | 21 | 92.56 | pI max (9.79) 110 a.a | Time (9, 164) |  | SET fold | Other folds | R/C |
| 28.59 | 18 | 93.17 | pI (6.53) |  | Fold |  |  | R/C |
| 26.60 | 20 | 93.20 | pI (9.60) | Time (6, 163) |  | SET fold | Other folds | R/C |
| 26.60 | 20 | 93.20 | pI (9.60) | Time (6, 163) |  | SET fold | Other folds | R/C |
| 28.72 | 18 | 93.44 | pI (6.54) |  | No_cluster | SET fold | Other folds | R/C |
| 25.80 | 21 | 93.60 | pI max (9.12) 155 a.a | Time (9, 164) |  | SET fold | Other folds | R/C |
| 28.94 | 18 | 93.89 | pI (6.53) |  | Ox | SET fold | Other folds | R/C |
| 26.00 | 21 | 94.01 | pI (6.55) | R/B | SPOUT fold | SET fold | Other folds | R/C |
| 30.01 | 17 | 94.02 | pI (9.93) | Time (7, 163) |  |  | Other folds | R/C |
