# Supplementary material for: Probabilistic Approach to Predicting Substrate Specificity of Methyltransferases
Source: PLoS Comput Biol. 2014 Mar 20;10(3):e1003514. doi: 10.1371/journal.pcbi.1003514 (PMC3961171; doi:10.1371/journal.pcbi.1003514)
Supplement: Table S4 — Description of MTase properties tested in the model. Beside properties described in the table, categorical property values were also used as independent properties with value true or false. Their names are: Ox, R/B, R/C, No cluster, Rossmann-like, SET, SPOUT, other fold, nucleus, nucleolus, mitochondrion, other localization. Those binary properties have 5 parameters. (DOC) [file pcbi.1003514.s008.doc]

**Table S4. Description of MTase properties tested in the model.**

| Property type | | | Properties | Number of parameters | Description |
| --- | --- | --- | --- | --- | --- |
| Fold | | | Rossmann-like | 11 | Structural fold of MTase catalytic domain (2). |
|  |  |  | SET |  |  |
|  |  |  | SPOUT |  |  |
|  |  |  | Other folds |  |  |
| Expression pattern | Expression cluster | | Ox | 11 | Expression clusters are derived from gene expression profiled during the Yeast Metabolic Cycle (YMC) (3), for genes with periodicity score > 1.96 sigma. The clusters are Ox (oxidative), R/C (reductive, charging), R/B (reductive, building) for. Non-periodic MTases are in “no cluster” group. |
|  |  |  | R/C |  |  |
|  |  |  | R/B |  |  |
|  |  |  | No cluster |  |  |
|  | Expression onset | | Time  2 intervals | 7 | The time of expression onset is taken from the YMC, which has 300 min period, so 0th min is equivalent to 300th min (3). For all MTases, expression time was used if they had periodicity score > 1.96, except for MTases with expression time ≥ 280th min or ≤ 16th min, periodicity score > 1.25 sigma was required (many genes with expression onsets in this time interval have very narrow expression profiles and thus receive lower periodicity scores (3, 4)). |
|  |  |  | Time  3 intervals | 11 |  |
| Localization | | | Nucleolus | 11 | Localization in the cell from Gene Ontology (5) (Inferred from Direct Assay (IDA) or Inferred from Electronic Annotation (IEA)) (as of 27^th^ January 2011). Other localizations include unknown localization category; localizations listed are mutually exclusive. |
|  |  |  | Nucleus AND not nucleolus |  |  |
|  |  |  | Mitochondrion AND not nucle-us |  |  |
|  |  |  | Other localizations |  |  |
| Isoelectric point | | pI | 2 pI intervals | 6 | pI was calculated for the whole protein, based on experimental pI values for individual amino acids taken from Lehninger et al (6). |
|  |  |  | 3 pI intervals | 10 |  |
|  |  | pI min | pI min  2 intervals | 7 | pI was calculated for parts of the protein, using sliding window ranging from 15 to 185 a.a. with the 15 a.a. step. pI min and pI max is defined as the a minimal or a maximal pI value for each protein and each window size. |
|  |  |  | pI min  3 intervals | 11 |  |
|  |  | pI max | pI max  2 intervals | 7 |  |
|  |  |  | pI max  3 intervals | 11 |  |
