# Supplementary material for: Probabilistic Approach to Predicting Substrate Specificity of Methyltransferases
Source: PLoS Comput Biol. 2014 Mar 20;10(3):e1003514. doi: 10.1371/journal.pcbi.1003514 (PMC3961171; doi:10.1371/journal.pcbi.1003514)
Supplement: Table S5 — Substrate specificity predictions for known MTases. (DOC) [file pcbi.1003514.s009.doc]

**Table S5. Substrate specificity predictions for known MTases.**

| MTase | Predicted substrate | | | Experimentally confirmed substrate | Prediction correct? |
| --- | --- | --- | --- | --- | --- |
|  | Protein | RNA | Other |  |  |
| YBL024W (TRM4) | 0.29 | **0.66** | 0.04 | RNA | Yes |
| YBR030W (RKM3) | **0.97** | 0.02 | 0.02 | Protein | Yes |
| YBR034C (HMT1) | **0.67** | 0.22 | 0.11 | Protein | Yes |
| YBR061C (TRM7) | 0.14 | **0.84** | 0.02 | RNA | Yes |
| YBR133C (HSL7) | **0.67** | 0.22 | 0.11 | Protein | Yes |
| YBR236C (ABD1) | 0.14 | **0.84** | 0.02 | RNA | Yes |
| YBR261C (TAE1) | **0.67** | 0.22 | 0.11 | Protein | Yes |
| YBR271W | **0.67** | 0.22 | 0.11 | Protein | Yes |
| YCL054W (SPB1) | 0.14 | **0.84** | 0.02 | RNA | Yes |
| YCR047C (BUD23) | 0.14 | **0.84** | 0.02 | RNA | Yes |
| YDL014W (NOP1) | 0.14 | **0.84** | 0.02 | RNA | Yes |
| YDL112W (TRM3) | **0.67** | 0.22 | 0.11 | RNA | No |
| YDL200C (MGT1) | 0.22 | **0.52** | 0.26 | Protein | No |
| YDL201W (TRM8) | 0.14 | **0.84** | 0.02 | RNA | Yes |
| YDR120C (TRM1) | 0.14 | **0.84** | 0.02 | RNA | Yes |
| YDR140W (MTQ2) | **0.67** | 0.22 | 0.11 | Protein | Yes |
| YDR198C (RKM2) | **0.97** | 0.02 | 0.02 | Protein | Yes |
| YDR257C (RKM4) | **0.97** | 0.02 | 0.02 | Protein | Yes |
| YDR410C (STE14) | 0.22 | **0.52** | 0.26 | Protein | No |
| YDR435C (PPM1) | 0.33 | 0.16 | **0.51** | Protein | No |
| YDR440W (DOT1) | 0.14 | **0.84** | 0.02 | Protein | No |
| YDR465C (RMT2) | **0.67** | 0.22 | 0.11 | Protein | Yes |
| YER091C (MET6) | 0.37 | 0.05 | **0.58** | Other | Yes |
| YER175C (TMT1) | 0.36 | 0.07 | **0.57** | Other | Yes |
| YGL050W (TYW3) | 0.22 | **0.52** | 0.26 | RNA | Yes |
| YGL136C (MRM2) | 0.14 | **0.84** | 0.02 | RNA | Yes |
| YGL192W (IME4) | 0.18 | **0.60** | 0.22 | RNA | Yes |
| YGR157W (CHO2) | 0.07 | 0.09 | **0.84** | Other | Yes |
| YHL039W (EFM1) | **0.97** | 0.02 | 0.02 | Protein | Yes |
| YHR070W (TRM5) | 0.14 | **0.84** | 0.02 | RNA | Yes |
| YHR109W (CTM1) | **0.97** | 0.02 | 0.02 | Protein | Yes |
| YHR119W (SET1) | **0.76** | 0.23 | 0.01 | Protein | Yes |
| YIL064W (SEE1) | **0.67** | 0.22 | 0.11 | Protein | Yes |
| YIL110W (HPM1) | **0.67** | 0.22 | 0.11 | Protein | Yes |
| YJL125C (GCD14) | 0.14 | **0.84** | 0.02 | RNA | Yes |
| YJL168C (SET2) | **0.76** | 0.23 | 0.01 | Protein | Yes |
| YJR073C (OPI3) | 0.07 | 0.09 | **0.84** | Other | Yes |
| YKR056W (TRM2) | 0.14 | **0.84** | 0.02 | RNA | Yes |
| YKR069W (MET1) | 0.37 | 0.05 | **0.58** | Other | Yes |
| YLL062C (MHT1) | 0.37 | 0.05 | **0.58** | Other | Yes |
| YLR137W | 0.36 | 0.07 | **0.57** | Protein | No |
| YLR172C (DPH5) | 0.37 | 0.05 | **0.58** | Protein | No |
| YLR186W (EMG1) | 0.14 | **0.84** | 0.02 | RNA | Yes |
| YLR285W (NNT1) | **0.67** | 0.22 | 0.11 | Protein | Yes |
| YML008C (ERG6) | 0.36 | 0.07 | **0.57** | Other | Yes |
| YML014W (TRM9) | 0.14 | **0.84** | 0.02 | RNA | Yes |
| YML110C (COQ5) | 0.36 | 0.07 | **0.57** | Other | Yes |
| YNL063W (MTQ1) | 0.14 | **0.84** | 0.02 | Protein | No |
| YOL093W (TRM10) | 0.14 | **0.84** | 0.02 | RNA | Yes |
| YOL096C (COQ3) | 0.36 | 0.07 | **0.57** | Other | Yes |
| YOL124C (TRM11) | 0.14 | **0.84** | 0.02 | RNA | Yes |
| YOL125W (TRM13) | 0.14 | **0.84** | 0.02 | RNA | Yes |
| YOL141W (PPM2) | **0.67** | 0.22 | 0.11 | RNA | No |
| YOR074C (CDC21) | 0.35 | 0.12 | **0.53** | Other | Yes |
| YOR201C (MRM1) | 0.14 | **0.84** | 0.02 | RNA | Yes |
| YOR239W (ABP140) | **0.67** | 0.22 | 0.11 | RNA | No |
| YPL030W (TRM44) | 0.14 | **0.84** | 0.02 | RNA | Yes |
| YPL157W (TGS1) | 0.14 | **0.84** | 0.02 | RNA | Yes |
| YPL208W (RKM1) | **0.97** | 0.02 | 0.02 | Protein | Yes |
| YPL266W (DIM1) | 0.14 | **0.84** | 0.02 | RNA | Yes |
| YPL273W (SAM4) | 0.37 | 0.05 | **0.58** | Other | Yes |
