# Supplementary material for: Probabilistic Approach to Predicting Substrate Specificity of Methyltransferases
Source: PLoS Comput Biol. 2014 Mar 20;10(3):e1003514. doi: 10.1371/journal.pcbi.1003514 (PMC3961171; doi:10.1371/journal.pcbi.1003514)
Supplement: Table S6 — Substrate specificity predictions for putative MTases. (DOC) [file pcbi.1003514.s010.doc]

**Table S6**. Substrate specificity predictions for putative MTases.

| MTase | Predicted substrate | | | Experimentally confirmed substrate | Prediction correct? |
| --- | --- | --- | --- | --- | --- |
|  | Protein | RNA | Other |  |  |
| YBR141C | 0.14 | **0.84** | 0.02 | - | - |
| YBR225W | 0.18 | **0.60** | 0.22 | - | - |
| YCL055W (KAR4) | 0.18 | **0.60** | 0.22 | - | - |
| YDR083W (RRP8) | 0.14 | **0.84** | 0.02 | - | - |
| YDR316W (OMS1) | 0.14 | **0.84** | 0.02 | Not protein | Yes |
| YGR001C (AML1) | **0.67** | 0.22 | 0.11 | - | - |
| YGR283C | 0.14 | **0.84** | 0.02 | Not protein | Yes |
| YHR207C (SET5) | **0.84** | 0.01 | 0.15 | Protein | Yes |
| YHR209W (CRG1) | 0.36 | 0.07 | **0.57** | Other | Yes |
| YIL096C | 0.14 | **0.84** | 0.02 | Not protein | Yes |
| YJL105W (SET4) | **0.76** | 0.23 | 0.01 | - | - |
| YJR129C | **0.67** | 0.22 | 0.11 | - | - |
| YKL155C (RSM22) | 0.14 | **0.84** | 0.02 | Not protein | Yes |
| YKL162C | 0.18 | **0.60** | 0.22 | - | - |
| YKR029C (SET3) | **0.77** | 0.13 | 0.11 | - | - |
| YLR063W | 0.14 | **0.84** | 0.02 | - | - |
| YMR209C | 0.14 | **0.84** | 0.02 | - | - |
| YMR228W (MTF1) | **0.67** | 0.22 | 0.11 | - | - |
| YMR310C | 0.14 | **0.84** | 0.02 | Not protein | Yes |
| YNL022C (RCM1) | 0.14 | **0.84** | 0.02 | - | - |
| YNL024C | **0.67** | 0.22 | 0.11 | - | - |
| YNL061W (NOP2) | **0.67** | 0.22 | 0.11 | - | - |
| YNL092W | 0.36 | 0.07 | **0.57** | Protein | No |
| YOR021C | **0.67** | 0.22 | 0.11 | Protein | Yes |
| YPL165C (SET6) | **0.76** | 0.23 | 0.01 | - | - |
