# Supplementary material for: Probabilistic Approach to Predicting Substrate Specificity of Methyltransferases
Source: PLoS Comput Biol. 2014 Mar 20;10(3):e1003514. doi: 10.1371/journal.pcbi.1003514 (PMC3961171; doi:10.1371/journal.pcbi.1003514)
Supplement: Text S1 — Supplementary text. (DOC) [file pcbi.1003514.s011.doc]

**SUPPORTING INFORMATION**

**Methods**

Eq. 2 in the main manuscript requires a value of the probability that an MTase has a certain property under the condition that it has a particular substrate type. We used two approaches to calculate this value, depending on whether the property in question was a continuous or a categorical variable. To calculate the value, we took the number of known MTases with a certain substrate and certain property and the number of all known MTases with the selected substrate type. Then (i) we assumed that the training set was a part of a bigger population and used hypergeometric probability distribution to estimate the probability for the whole population, (ii) additionally, for continuous properties we smoothed the probability function to avoid rapid changes between intervals.

Two applied approaches:

(i) Since MTases with known substrates are only a representation of the whole population of MTases, we do not use the probabilities *P*(property|substrate_i_) computed for the set of known MTases, but rather estimate the true value for the whole population. Estimating these probabilities is especially crucial for combinations of properties and substrates that do not exist among known MTases, that is for which sample probabilities equal 0. To estimate *P*(property|substrate_i_) needed in Eq. 1 in the main manuscript, we assume that the population size is 1000 and determine *n* such that it minimizes the absolute value of:

$\sum_{i=0}^{n-1} P(k|i)-\sum_{i=n+1}^{1000} P(k|i)$, (ЕS1)

where *k* is the observed number of MTases with a given combination of features and substrate speciﬁcity, *i* is the number of such MTases in the population of 1000 and *P*(*k*|*i*) is the probability of observing *k* such MTases while sampling a population of 1000 containing *i* of them, computed using hypergeometric probability distribution.

(ii) In the present case, the only continuous properties considered were pI and time of expression onset. We observed that for both of these properties and for each substrate speciﬁcity, their domain can be divided into several intervals, within each their probability distribution functions have similar values. Therefore, we decided to model these probability distributions as a step function, smoothed to avoid rapid changes of probability between intervals. After some experimentation, we decided to use a high-order even exponential as a smoothing function, speciﬁcally $e^{-f(x)^{44}}$ , where *f*(*x*) is a linear function of *x*. Thus, the whole probability distribution for continuous variables would be a linear combination of several exponential terms, each corresponding to a chosen interval, with weights derived from average probabilities within a given interval and with function *f* determined by the beginning and ends of a given interval. Examples of speciﬁc functions used are given in ﬁgures (see Fig. S3 and S4).

**Threshold optimization**

We optimized thresholds for each model by likelihood maximization using the Powell method [1]. The starting points for optimization were quantiles of all MTases values chosen as suitable for each property variant, e.g. pI, divided into two intervals, had the median as the starting point.

**Tested sets of properties**

We tested all combinations of the properties described in Table S4 except combinations including:

- any two of the pI, pI min and pI max,
- both Localization and any of the Nucleus, Nucleolus, Mitochondrion or Other localization properties,
- both Fold and any from the binary properties Rossman-like, SET, SPOUT, Other fold,
- both Expression cluster and any from Ox, R/B, R/C or No cluster binary properties.

Versions of the same property (*pI* or *time*) divided into intervals diﬀerently were also not tested within the same model.

**Akaike Information Criterion (AIC)**

Akaike Information Criterion [7] was designed to help achieve a balance between model accuracy and complexity. It is given by the equation:

$AIC=\text{2k}-\text{2ln}(L)$, (ES2)

where *k* is the number of parameters in the model and *L* is the maximized value of the likelihood function for the estimated model. As seen, the penalty factor based on the number of parameters is added to avoid the eﬀect of a more complex model always better ﬁtting the data. The number of parameters of a model was calculated by summing the number of parameters from each property (Table S4) plus adding 2 to account for prior probabilities (2 independent parameters are needed to describe *a priori* probabilities of protein, RNA and other substrate speciﬁcity).

**SUPPORTING INFORMATION REFERENCES**

1. Powell MJD (1964) An efficient method for finding the minimum of a function of several variables without calculating derivatives. Computer Journal 7: 155-162.

2. Wlodarski T, Kutner J, Towpik J, Knizewski L, Rychlewski L, et al. (2011) Comprehensive structural and substrate specificity classification of the Saccharomyces cerevisiae methyltransferome. PLoS One 6: e23168.

3. Tu BP, Kudlicki A, Rowicka M, McKnight SL (2005) Logic of the yeast metabolic cycle: temporal compartmentalization of cellular processes. Science 310: 1152-1158.

4. Rowicka M, Kudlicki A, Tu BP, Otwinowski Z (2007) High-resolution timing of cell cycle-regulated gene expression. Proc Natl Acad Sci U S A 104: 16892-16897.

5. Ashburner M, Ball CA, Blake JA, Botstein D, Butler H, et al. (2000) Gene ontology: tool for the unification of biology. The Gene Ontology Consortium. Nat Genet 25: 25-29.

6. Lehninger A, Nelson DL, Cox MM (2000) Principles of Biochemistry. New York: Worth Publisher.

7. Akaike H (1974) A new look at the statistical model identification. System identification and time-series analysis. IEEE Trans Automatic Control AC-19: 716-723.
